# Variant-selective stereopure oligonucleotides protect against pathologies associated with *C9orf72*-repeat expansion in preclinical models

**DOI:** 10.1038/s41467-021-21112-8

**Published:** 2021-02-08

**Authors:** Yuanjing Liu, Jean-Cosme Dodart, Helene Tran, Shaunna Berkovitch, Maurine Braun, Michael Byrne, Ann F. Durbin, Xiao Shelley Hu, Naoki Iwamoto, Hyun Gyung Jang, Pachamuthu Kandasamy, Fangjun Liu, Kenneth Longo, Jörg Ruschel, Juili Shelke, Hailin Yang, Yuan Yin, Amy Donner, Zhong Zhong, Chandra Vargeese, Robert H. Brown

**Affiliations:** 1 Wave Life Sciences Ltd., Cambridge, MA USA; 2 Department of Neurology, University of Massachusetts, Worcester, MA USA

**Keywords:** Antisense oligonucleotide therapy, Pharmaceutics, Amyotrophic lateral sclerosis, Amyotrophic lateral sclerosis

## Abstract

A large G_4_C_2_-repeat expansion in *C9orf72* is the most common genetic cause of amyotrophic lateral sclerosis (ALS) and frontotemporal dementia (FTD). Neuronal degeneration associated with this expansion arises from a loss of C9orf72 protein, the accumulation of RNA foci, the expression of dipeptide repeat (DPR) proteins, or all these factors. We report the discovery of a new targeting sequence that is common to all *C9orf72* transcripts but enables preferential knockdown of repeat-containing transcripts in multiple cellular models and C9BAC transgenic mice. We optimize stereopure oligonucleotides that act through this site, and we demonstrate that their preferential activity depends on both backbone stereochemistry and asymmetric wing design. In mice, stereopure oligonucleotides produce durable depletion of pathogenic signatures without disrupting protein expression. These oligonucleotides selectively protect motor neurons harboring *C9orf72*-expansion mutation from glutamate-induced toxicity. We hypothesize that targeting *C9orf72* with stereopure oligonucleotides may be a viable therapeutic approach for the treatment of *C9orf72*-associated neurodegenerative disorders.

## Abstract (web summary)

*C9orf72* expansion mutations are the most common genetic cause of ALS and FTD, which have limited therapies. The authors generate stereopure oligonucleotides that selectively deplete expansion-containing transcripts and protect against expansion-associated pathologies in preclinical models.

## Introduction

A large hexanucleotide-repeat (G_4_C_2_) expansion in the first intron or promoter region of *C9orf72* is the most common cause of familial amyotrophic lateral sclerosis (ALS) and frontotemporal dementia (FTD)^1–3^. ALS is a fatal neuromuscular disease resulting from the degeneration of motor neurons in the brain and spinal cord^4^. FTD is the second most common form of dementia after Alzheimer’s disease, resulting from neurodegeneration in the frontal and anterior temporal lobes of the brain^4^. The discovery of *C9orf72*-repeat expansion as a common autosomal dominant cause for these two diseases suggests that they are manifestations of a continuous clinical spectrum, denoted herein as C9-FTD/ALS. There are no effective treatments available for FTD. While there are treatments approved for ALS (http://www.alsa.org/als-care/fda-approved-drugs.html), the efficacies are modest, and more effective treatments are needed.

Multiple molecular pathologies have been associated with C9-FTD/ALS. The expansion-containing allele is transcribed bi-directionally^5–7^, and the resulting sense and antisense RNAs accumulate into nuclear foci^1,5–11^, which can sequester RNA-binding proteins and impair their ability to perform their normal regulatory roles^12,13^. Accumulation of repeat-containing transcripts can produce motor neuron deficits and C9-ALS/FTD-like symptoms in animal models^9–11,14^. The repeat expansion may lead to haploinsufficiency for C9orf72 protein^11,15–17^. Although depletion of *C9orf72* in mice does not lead to overt neurodegenerative phenotypes^11,18^, *C9orf72*-null mice exhibit age-related social abnormalities and mild deficits in rotarod-assays, as well as inflammatory phenotypes that could contribute to disease^11^. Furthermore, in motor neurons derived from human-induced pluripotent stem cells (iPSCs), the repeat expansion leads to partial loss of C9orf72 function, disrupting vesicular trafficking and causing glutamate-induced excitotoxicity^15^. The repeat expansion also leads to the generation and accumulation of neurotoxic dipeptide-repeat proteins (DPRs)^5,7,9–11,14,19,20^. These peptides are produced from repeat-associated non-ATG (RAN) translation of sense and antisense transcripts. Similar to the accumulation of transcripts, these DPRs can produce motor neuron deficits and C9-ALS/FTD-like phenotypes in animal^9–11,21–23^ and cellular models^24^. Because the relative contributions of these mechanisms to neurodegeneration in C9-ALS/FTD are unclear, we sought to develop oligonucleotides that promote RNase H-mediated degradation of pathogenic C9orf72 transcriptional variants that also preserve C9orf72 protein expression and disrupt the production of DPRs. Oligonucleotides can penetrate the nucleus and have the potential to engage both newly transcribed pre-mRNAs as well as those sequestered in RNA foci. Others have reported that 2′-O-methyl (2′-OMe)-^25^ or 2′-O-(2-methoxyethyl) (2′-MOE)-modified^8,11^ oligonucleotides targeting the G_4_C_2_ expansion in *C9orf72* can selectively deplete expansion-containing transcripts in models for C9-ALS/FTD. In C9^450B^ BAC transgenic mice, which express truncated *C9orf72* RNAs containing ~450 repeats, select oligonucleotides retained expression of exon 1b-containing transcripts, favorably impacted some pathogenic signatures of disease, and nominally alleviated age-dependent anxiety and cognition phenotypes^11^. These reports provide evidence that oligonucleotides that mediate selective knockdown of repeat-containing transcripts may be viable therapeutics for the treatment of C9-ALS/FTD.

We recently reported a synthetic chemistry platform that enables the production of stereopure oligonucleotides^26^. Oligonucleotides typically incorporate chiral phosphorothioate (PS) substitutions in the phosphodiester (PO) backbone to improve their metabolic stability and cellular uptake^27^. Traditional synthetic methods generate stereoisomeric mixtures composed of hundreds of thousands of PS-modified molecules, each with distinct stereochemistry and consequently, distinct pharmacologic properties^27^. Our chemistry platform has enabled the production of stereopure PS-modified oligonucleotides with controlled placement of *S*p and *R*p PS linkages in the backbone. We have demonstrated that the position of *S*p and *R*p linkages can impact the activity of an oligonucleotide, yielding more durable RNase H-mediated knockdown in mice compared with stereorandom analogs^26^.

We apply our platform to generate stereopure oligonucleotides, exemplified by C9orf72-631, that potently and preferentially knockdown repeat-containing *C9orf72* transcripts in cellular and mouse models through a target sequence that is common to all transcripts. Potent and selective activity at this site is enabled by the combination of backbone stereochemistry and asymmetric wing chemistry. Importantly, C9orf72-631 preserves C9orf72 protein expression and durably diminishes the pathological signatures of C9-ALS/FTD in mice.

## Results

**Fig. 1:**
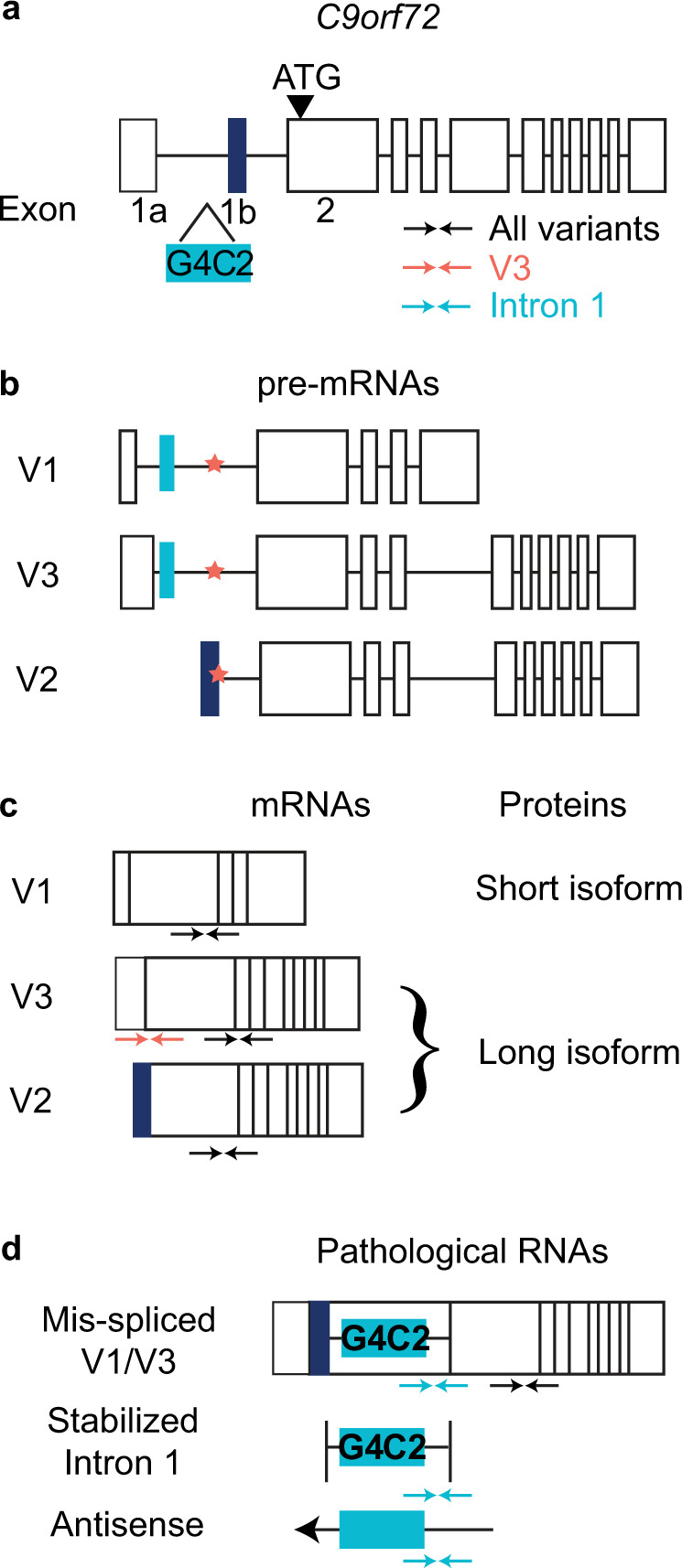
Schematic representation of C9orf72 gene structure, RNA, and protein products, and the strategy for detecting C9orf72 transcripts. **a** *C9orf72* genomic structure is illustrated; boxes represent exons. Exon 1a and 1b (navy), which do not encode protein, are alternative first exons, and exon 2 contains the translational start site (ATG, black triangle). The G_4_C_2_-repeat expansion (aqua) is in intron 1, upstream of exon 1b. Approximate position of primers used to detect various transcripts in Taqman assays are illustrated with arrows, including primers that span the exon 2–exon 3 junction (black) that detects all transcripts, primers that span the exon 1a–exon 2 junction (coral) that are specific to V3, and primers complementary to intron 1 (aqua) that detect all intron 1-containing and expansion-containing variants. **b** pre-mRNAs corresponding to V1–V3 are illustrated. The coral star indicates SS1b. **c** Mature mRNAs for all variants and the proteins they encode are shown. Primers for all variants detect all three mature mRNAs, whereas the V3 primers are specific to V3. Intron 1 primers do not detect any mature mRNAs. **d** Pathogenic RNA by-products resulting from the G_4_C_2_-repeat expansion in intron 1 are shown. The location of the G_4_C_2_-repeat expansion (aqua) is shown in sense and antisense RNAs. Intron 1 primers detect all pathological RNAs, and the all variants primers will detect any mis-spliced V1/V3 transcripts that include exon 2. SS1b Splice site 1b, V variant.

*C9orf72* contains multiple transcriptional start and polyadenylation sites^28^, which normally lead to the production of three transcriptional variants, denoted V1–V3 (Fig. 1); V2 is the most prevalent variant, and V1 is the least prevalent^29^. V1 encodes a short isoform of C9orf72 protein, while V3 and V2 encode the same long isoform but incorporate distinct non-coding first exons (1a or 1b, respectively). The disease-causing G_4_C_2_-repeat expansion in intron 1 leads to increased expression of exon 1a-containing variants (V1 and V3)^30^, ineffective splicing, the stabilization of repeat-containing transcripts^31^ and the generation of antisense transcripts^5,6^. The expansion can also decrease C9orf72 protein expression^15^ and lead to the production of DPRs from expansion-associated sense and antisense transcripts^19^. Our goal is to selectively eliminate variants impacted by the hexanucleotide repeat expansion. We expect this strategy to decrease expression of pathogenic RNAs and DPRs, as well as to spare most C9orf72 protein, and thereby address most of the major molecular mechanisms that are believed to underlie C9-ALS/FTD.

### Identification of SS1b at the exon 1b–intron 1 junction

To identify a targeting site with potential for achieving potent and selective depletion of V1 and V3, we performed a primary screen assessing stereorandom oligonucleotides for their ability to promote RNase H-mediated knockdown of a *C9orf72*-luciferase reporter gene in Cos-7 cells (Supplementary Fig. 1a–c). These oligonucleotides, called gapmers, are complementary to *C9orf72* mRNA spanning a 1-kb region encompassing intron 1 (Supplementary Table 1). We identified a sequence near the exon 1b–intron junction that yielded efficient knockdown of the luciferase signal (Supplementary Fig. 1c). To validate this activity in a more physiologically relevant model and to assess the impact of targeting this sequence on various *C9orf72* transcripts, we retested the activity of a subset of oligonucleotides in C9-ALS iPSC-derived motor neurons (denoted ALS motor neurons, Supplementary Fig. 1d–g). C9orf72-109, an oligonucleotide targeting exon 2 designed to deplete all *C9orf72* transcripts, showed no preference for V3 (Supplementary Fig. 1f, g). Of the oligonucleotides tested, C9orf72-135 was the most potent against V3, depleting levels to 0.59-fold of normal (Supplementary Fig. 1f). In the presence of C9orf72-135, expression of non-repeat-containing transcripts was preserved at 0.78-fold of normal (Supplementary Fig. 1g). Since V3 transcripts represent only ~3.8% of *C9orf72* transcripts and it is depleted to a greater extent than all transcripts (Supplementary Fig. 1d–g), these data illustrate that some preferential activity is achievable through this sequence even with an unoptimized oligonucleotide.

Next, we performed a microwalk in the vicinity of C9orf72-135 to identify the best targeting sequence (Supplementary Fig. 1h). Once again, C9orf72-135 yielded the most efficient knockdown in the C9-reporter system. Finally, we confirmed C9orf72-135’s activity in an additional series of cellular disease models: C9-ALS-iPSC-derived cortical neurons (denoted ALS cortical neurons), C9-ALS patient-derived fibroblasts and primary neurons from C9BAC transgenic mice^29^ (Supplementary Fig. 2a–e). In ALS cortical neurons, C9orf72-135 and C9orf72-109 depleted V3 transcripts in a dose-dependent manner (Supplementary Fig. 2b). Although C9orf72-135 also depleted the expression of all transcripts, it was far less potent than C9orf72-109 (Supplementary Fig. 2c). As with ALS motor neurons, V3 transcripts represent only ~3.8% of all transcripts in these cells (Supplementary Fig. 2a), indicating once again that some preferential activity is achievable through this sequence even with an unoptimized oligonucleotide. In ALS patient-derived fibroblasts and in C9BAC primary neurons, C9orf72-135 showed a slight preference for V3 compared with all variants (Supplementary Fig. 2d, e). In multiple in vitro model systems, C9orf72-135, which is an unoptimized oligonucleotide that targets a sequence at the exon 1b–intron 1 junction that is common to all *C9orf72* transcripts, yields preferential knockdown of exon 1a-containing transcripts. We call the sequence targeted by C9orf72-135 Splice Site-1b (SS1b).

### Optimization enhances potency and preferential activity

**Fig. 2:**
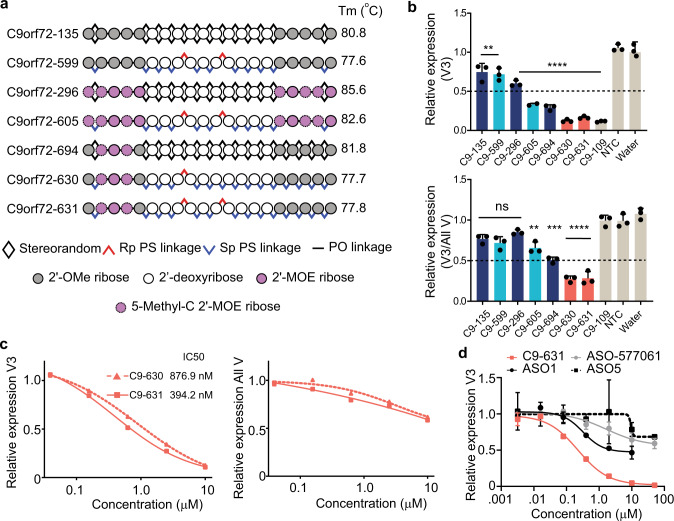
Backbone stereochemistry and asymmetric 2′-ribose modifications enhance the potency and preferential activity of oligonucleotides for repeat-containing C9orf72 transcripts. **a** Schematic representation of oligonucleotides used to explore the structure–activity relationship for preferential targeting of V3. Measured melting temperatures (*T*_m_) for the oligonucleotide-C9 surrogate RNA heteroduplexes are indicated. **b** Relative expression of V3 (top) or the ratio of V3 to all variants (bottom) with respect to *HPRT* in human ALS motor neurons after gymnotic treatment with 10 µM of the indicated oligonucleotides (stereorandom, navy; stereopure, aqua; stereopure with asymmetric wing chemistry, coral; controls, beige). Dotted horizontal line denotes 0.5-fold expression level for V3. Data are presented as mean ± SD, *n* = 3. One-way ANOVA with Dunnett’s multiple comparison test. ****P < 0.0001, ***P < 0.001, **P < 0.01, ns non-significant. **c** Relative expression of the indicated *C9orf72* transcript (V3, left; All variants, right) with respect to *HPRT* in human ALS motor neurons treated with increasing concentrations (0.001–10 µM) of C9orf72-630 or C9orf72-631. Lines represent four-parameter least-squares fit. Half-maximal inhibitory concentrations (IC_50_s) for V3 are indicated (*n* = 2). **d** Relative expression of V3 with respect to *HPRT* in human ALS motor neurons treated with increasing concentrations (3 nM–50 μM) of the indicated oligonucleotide under gymnotic conditions. Lines represent four-parameter non-linear fit of data. Data are presented as mean ± SD, *n* = 3. ASO1 was toxic to cells at concentrations >10 μM, precluding activity evaluations at high concentrations. Source data, including exact *P* values, are provided as a Source Data file. NTC non-targeting control; All V All variants, PS phosphorothioate, PO phosphodiester, C cytosine, OMe O-methyl, MOE methoxyethyl, V variant.

In our prior work^26^, we demonstrated that a stereopure oligonucleotide with defined 3′-*S*p*S*p*R*p-5′ (denoted hereafter SSR) PS-backbone stereochemistry can increase RNase H activity in vitro and in vivo compared with oligonucleotides based on industry standard chemistry [e.g., gapmers containing fully PS-modified stereorandom backbones with 2′-O-methoxyethyl (MOE)-modified wings]^32^. Based on these findings, we tested whether we could improve the potency and preferential activity of C9orf72-135 with backbone stereochemistry or other chemical modifications. First, we assessed the impact of SSR backbone stereochemistry with 2′-OMe-modified wings in C9orf72-599 and random stereochemistry with industry standard 2′-MOE-wing chemistry in C9orf72-296 (Fig. 2a, Supplementary Fig. 3a). Neither stereochemistry nor wing chemistry alone yielded better than 50% knockdown of the targeted V3 transcripts in ALS motor neurons (Fig. 2b).

To optimize for potency and preferential activity against V3 transcripts, we investigated the structure–activity relationship (SAR) among backbone stereochemistry and 2′-sugar modifications in the wings of oligonucleotides under free-uptake conditions (i.e., in the absence of transfection reagents) in ALS motor neurons. We found that the combination of SSR backbone stereochemistry with 2′-MOE-wing chemistry, in C9orf72-605, improved the potency of the oligonucleotide against V3 (knockdown to 0.33-fold of normal expression) while decreasing the ratio of V3 to all variants (Fig. 2b, Supplementary Fig. 3b). SAR investigations revealed a permutation containing distinct patterns of chemical modifications in the 5′- and 3′-wings called asymmetric wing chemistry. The most effective permutations contained 2′-OMe and 2′-MOE modifications in the 5′-wing and 2′-OMe modifications in the 3′-wing. The combination of SSR backbone stereochemistry with asymmetric wing chemistry in C9orf72-630 and C9orf72-631 yielded the most potent knockdown of V3 transcripts (0.12- and 0.13-fold of normal, respectively) while dramatically decreasing the ratio of V3 to all variants (Fig. 2b, Supplementary Fig. 3b). Similar trends were observed for these oligonucleotides in ALS cortical neurons (Supplementary Fig. 3c–e). C9orf72-630 (IC_50_ = 876.9 nM) and C9orf72-631 (IC_50_ = 394.2 nM) dose-dependently knocked down V3 transcripts in ALS motor neurons (Fig. 2c, left) and were preferentially active against V3 compared with all variants at all concentrations tested (Fig. 2c, right). We confirmed that these oligonucleotides were active in RNase H-mediated cleavage assays (Supplementary Fig. 3g), and their activity in these biochemical assays tracked with cellular data. We also show that a stereopure oligonucleotide lacking the SSR motif, C9orf72-879, was only weakly active, indicating that the SSR motif is necessary for enhanced RNase H activity. Importantly, C9orf72-630 and C9orf72-631 both feature a combination of SSR stereochemistry and asymmetric wing design.

Next, we compared our optimized oligonucleotides to other published oligonucleotides^8,11,30,33^. ASO-577061, which targets intron 1 in the vicinity of the repeat expansion and has been reported to yield selective knockdown of repeat-containing *C9orf72* transcripts in various cellular^8,30^ and mouse^11^ models. In ALS motor neurons under free-uptake conditions with 10 μM oligonucleotide, ASO-577061 was less active against V3 than C9orf72-630 or C9orf72-631 (retaining 0.66-fold V3 expression compared with 0.12-fold and 0.16-fold, respectively, Supplementary Fig. 3h), and it was not active enough under these conditions to evaluate selectivity. C9orf72-630 and C9orf72-631 effectively decreased V3 transcripts (*P* < 0.0001, one-way ANOVA), and significantly decreased the ratio of V3 to all variants (*P* < 0.0001, one-way ANOVA). In a related experiment, we compared C9orf72-631 to additional previously published oligonucleotides. ASO1 has been reported to deplete repeat-containing transcripts in cellular^8^ and mouse^11^ models, and ASO5 has been reported to deplete repeat-containing transcripts in a mouse model^11^. In ALS motor neurons, C9orf72-631 was more potent against V3 than all other oligonucleotides tested (Fig. 2d); ASO1, the next most potent oligonucleotide, was toxic at high concentrations, whereas ASO-577061 and ASO5 exhibited nominal activity. Thus, oligonucleotides designed with a combination of backbone stereochemistry and asymmetric wings direct more potent activity than other published oligonucleotides.

### Selective oligonucleotides do not displace splicing machinery from the exon1b junction

It was surprising to us that we could achieve preferential knockdown of expansion-containing transcripts by targeting SS1b, which is common to all pre-mRNAs (Fig. 1b). To investigate the source of this preferential activity, we measured oligonucleotide–RNA heteroduplex thermal melting temperatures (*T*_m_s) (Fig. 2a). Heteroduplex formation is necessary for RNase H-mediated activity. In general, thermal stability of a heteroduplex affects oligonucleotide activity, with increasing stability yielding increased activity^27^. However, ranking these oligonucleotides based on their activity (potency and preferential activity: C9orf72-631, −630 > −694, −605 > −296 > −599, −135) is not consistent with their heteroduplex stability as assessed by *T*_m_ (C9orf72-296 > −605 > −694 > −135 > −631, −630, −599), indicating that there must be another explanation for the observed activities.

**Fig. 3:**
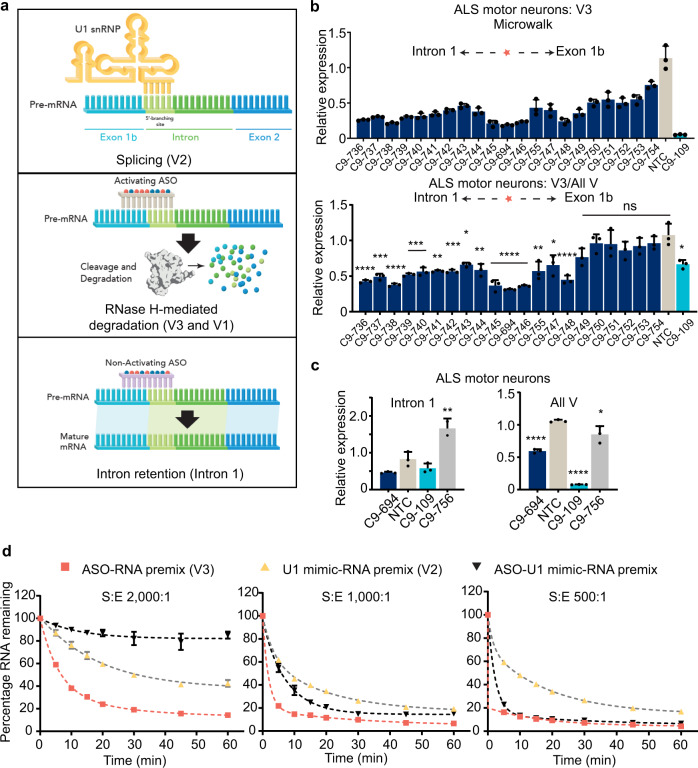
Oligonucleotides preferentially target transcripts that are not protected by the splicing machinery. **a** Schematic illustration of selectivity model. When the splicing machinery binds to SS1b (Splicing), the resulting transcription produces V2. An RNase H-active oligonucleotide (RNase H-mediated degradation) will selectively bind non-V2 variants and promote their degradation. An RNase H-inactive oligonucleotide with high *T*_m_ (Intron retention) will displace the splicing machinery yet leave the unspliced RNA intact, resulting in the accumulation of intron 1-containing transcripts. **b** Relative expression of V3 (top) and ratio of V3: all variants (bottom) with respect to *HPRT* in human ALS iPSC-derived motor neurons treated with 10 μM of the indicated oligonucleotide. The coral star marks the location of SS1b. Data are presented as mean ± SD, *n* = 3. One-way ANOVA with Dunnett’s multiple comparison test. ****P < 0.0001, ***P < 0.001, **P < 0.01, *P < 0.05. **c** Relative expression of intron-1-containing transcripts (left) and all variants (right) with respect to *HRPT* in human ALS motor neurons treated with 10 μM of the indicated oligonucleotide. Note that the *y* axes for the two graphs are different. Data are presented as mean ± SD, *n* = 3. One-way ANOVA with Dunnett’s multiple comparisons test with comparison to NTC ****P < 0.0001, **P < 0.01 (Intron 1 C9-756 *P* = 0.002), *P < 0.05 (All V C9-756 *P* = 0.0135). **d** Percentage of full-length surrogate RNA remaining after heteroduplex formation with oligonucleotide–RNA pre-mix (V3), U1 RNA mimic-RNA pre-mix (V2), or oligonucleotide, U1 RNA mimic and surrogate pre-mix and treatment with RNase H in vitro with respect to time. Data are presented as mean ± SEM, *n* = 3. Lines depict 2-phase decay for least-squares fit. The substrate (S) to enzyme (E) ratio for each panel is shown. Source data, including exact *P* values for panel **b**, are provided as a Source Data file. snRNP small nuclear ribonuclearprotein, ASO antisense oligonucleotide, V variant.

To investigate the mechanism by which oligonucleotides that interact with SS1b achieve selectivity for V3, we considered known differences among *C9orf72* variants. For V2 only, splicing machinery, including U1 small nuclear ribonucleoprotein (snRNP), is expected to interact with this region at the 5′-splice site^34^. We hypothesize that the presence of the U1 snRNP and other components of the splicing machinery in this region protects V2, and that the oligonucleotides that mediate selective activity through this site bind to it with sufficient potency to promote RNase H-mediated cleavage of *C9orf72* variants that are not already bound by splicing factors but not with sufficient potency to displace the splicing machinery (Fig. 3a).

To test this hypothesis, we performed a series of biochemical and cellular experiments. First, we performed a microwalk, targeting sequences spanning the exon 1b–intron 1 boundary in ALS motor neurons with oligonucleotides based on C9orf72-694 that differ from each other only in sequence. If the splicing machinery at the exon 1b–intron 1 boundary protects V2, then shifting the oligonucleotide away from this boundary should decrease selectivity (Supplementary Fig. 4). Among the oligonucleotides tested, C9orf72-694, which binds to SS1b, and oligonucleotides that bind to regions immediately adjacent to SS1b (C9orf72-745 and C9orf72-746) are the most potent against V3 and show the most preference for this variant compared with all variants (Fig. 3b). As the targeting sequence moves away from SS1b, especially into exon 1b, selectivity for V3 is lost.

Next, we tested an RNase H-inactive oligonucleotide, C9orf72-756, which is complementary to this region and has a high measured *T*_m_ (>95 °C). Because C9orf72-756 forms a stable RNA–RNA duplex, it should effectively compete with the splicing machinery for SS1b without promoting RNase H-mediated activity; therefore, it should disrupt splicing and increase expression of transcripts that retain intron 1. In ALS motor neurons, C9orf72-694 led to the expected degradation of intron 1-containing transcripts (0.46-fold of normal, Fig. 3c). C9orf72-756 did not substantially impact the expression of all variants but increased the expression of intron 1-containing transcripts to 1.66-fold of normal (Fig. 3c), indicating that normal splicing at this site has been disrupted.

Finally, in biochemical assays, we assessed the ability of C9orf72-631 to promote RNase H-mediated degradation of a complementary C9orf72 RNA in conditions that mimic those present in cells for various transcripts (Supplementary Fig. 5). In the first scenario modeling V3, the RNA surrogate is pre-mixed with oligonucleotide prior to the addition of a short U1 mimic RNA and RNase H. Under these conditions, the RNA surrogate is rapidly degraded regardless of the amount of RNase H (Fig. 3d). In the second scenario modeling V2, the RNA surrogate is pre-mixed with a short U1 mimic RNA prior to the addition of oligonucleotide and RNase H. Under these conditions, the U1 mimic protects the RNA from degradation independent of RNase H concentration. In a third scenario, we pre-mix the RNA surrogate with both the U1 mimic and the oligonucleotide. Under these conditions, the U1 mimic protects the RNA from degradation only when RNase H is limiting (Fig. 3d, compare left panel to center and right panels). These data suggest that even a weak U1 mimic, with low *T*_m_ (25.8 °C) that is only partially complementary to the C9orf72 RNA, can protect the RNA from RNase H-mediated degradation in the presence of an oligonucleotide with a moderate *T*_m_ (77.7 °C) if it is pre-bound to the RNA, supporting our hypothesis that C9orf72-631 does not displace U1 snRNP or the splicing machinery from V2. Taken together, these data support the premise that C9orf72-631 and other selective oligonucleotides that act through SS1b are not sufficiently potent to displace splicing machinery from V2 but are potent enough to direct RNase H-mediated knockdown of accessible variants.

### C9orf72-631 diminishes pathogenic features associated with G_4_C_2_-repeat expansion in mice

2′-MOE chemical modifications^35^ and *S*p PS stereochemistry in the backbone^26^ are known to protect oligonucleotides from degradation. To assess the relative stability of our oligonucleotide panel prior to testing them in vivo, we measured their stability ex vivo in mouse brain homogenates (Supplementary Fig. 6a). These experiments demonstrate that while oligonucleotides with asymmetric wings are destabilized compared to those with symmetric, 2′-MOE-modified wings (compare C9orf72-694 to C9orf72-296), the introduction of predominately *S*p PS backbone stereochemistry was sufficient to restore stability (compare C9orf72-631 to C9orf72-296).

**Fig. 4:**
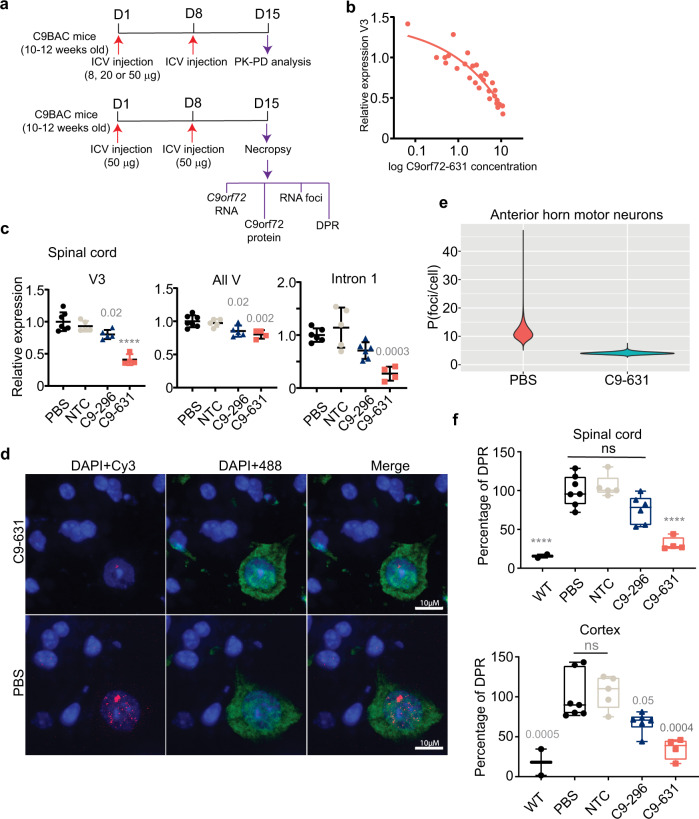
Stereopure oligonucleotides lead to the preferential reduction of repeat-containing transcripts, RNA foci, and DPR proteins in C9BAC mice. **a** Schematic representation for dosing regimens: single-dose escalation study for panel **b** (top), and 2-week study for panels **c**–**f** (bottom). For both regimens, animals received the same dose in both ICV injections on days (D) 1 and 8. Assays performed at necropsy are indicated. **b** Relative V3 expression (standardized to *Hprt*) is plotted with respect to the log concentration of C9orf72-631 in the spinal cord. **c** Relative expression levels for V3 (left) all variants (All V, middle) and intron 1-containing transcripts (right) in spinal cord are shown for the indicated oligonucleotide. Expression is standardized to *Hprt*, individual points indicate data from one mouse, Data are presented as mean ± SD. One-way ANOVA with Dunnett’s multiple comparisons test. Exact *P* values are indicated on graph, ****P < 0.0001. **d** Representative images from the evaluation of four mice per treatment group, showing spinal cords of mice treated with C9orf72-631 (top) or PBS (bottom) and stained for RNA foci (Cy3-labeled probe, red), nuclei (DAPI, blue), and motor neurons (anti-NeuN, green). 10 μm scale bars are shown. **e** Quantification of RNA foci per cell in anterior horn motor neurons in mice treated with PBS (fuschia) or C9orf72-631 (aqua) (Supplementary Fig. 7). **f** Percentage of RAN-translated DPR protein (polyGP) in neurons from the spinal cord (top) or cortex (bottom) is shown for the indicated treatment (WT untreated *n* = 2; PBS treated *n* = 7; C9orf72-296-treated *n* = 6; C9orf72-631 *n* = 4). Boxes delineate minima and maxima with horizontal lines denoting means. One-way ANOVA with Dunnett’s multiple comparisons test. Exact *P* values are indicated on graphs, ****P < 0.0001, ns non-significant. Source data are provided as a Source Data file. ICV intracerebroventricular, PBS phosphate-buffered saline, NTC non-targeting control, All V all variants, DPR dipeptide repeat, WT wild-type mouse.

The activity of oligonucleotides in cellular systems is not always predictive of their activity in vivo. To assess whether the activity of oligonucleotides that we optimized in cellular models under free-uptake conditions translates to an in vivo model, we assessed the activity of a subset of our oligonucleotides in C9BAC mice (Supplementary Fig. 6b)^18^. To determine the appropriate dose for C9BAC mice, we evaluated the PK–PD relationship for C9orf72-631 in a single dose-escalation study (Fig. 4a, b). C9orf72-631 led to dose-dependent activity against V3 in the spinal cord with an excellent correspondence between PK and PD (Fig. 4b). In a separate PK study (Supplementary Fig. 6c–e), C9orf72-631, administered as a single 100 μg dose, penetrated and persisted in the CNS for more than 1 month (spinal cord *t*_1/2_ = 39.1 days; cortex *t*_1/2_ = 44.2 days), which suggests C9orf72-631 may lead to durable transcript knockdown.

To assess the activity of our oligonucleotides in vivo, we evaluated their potency and preferential activity 2 weeks after ICV injection (Fig. 4a, bottom). V3 decreased to 0.41-fold of controls in the spinal cord (*P* < 0.0001, one-way ANOVA) and 0.67-fold in the cortex (*P* = 0.0079, one-way ANOVA) in animals administered C9orf72-631 (Fig. 4c, Supplementary Fig. 7a). In these animals, the ratio of V3 to all variants decreased compared with controls (Supplementary Fig. 7a), and intron 1-containing variants decreased to 0.27-fold of controls in spinal cord (Fig. 4c). By comparison, C9orf72-296 (the stereorandom oligonucleotide) was less potent against V3 (Fig. 4c, Supplementary Fig. 7a), decreasing V3 only to 0.80-fold of controls in the spinal cord and not significantly effecting V3 in cortex (*P* = 0.0678, one-way ANOVA). C9orf72-631 outperformed C9orf72-296, which targets the same sequence but lacks optimized chemistry and stereochemistry, indicating that the optimization we performed under free-uptake conditions in cells translated to mouse models.

Next, we assessed whether the oligonucleotides impacted pathogenic signatures of C9-ALS/FTD, including the accumulation of sense RNA foci and the amount of DPR proteins. We quantified sense RNA foci in motor neurons of the anterior horn of the spinal cord (Supplementary Fig. 7b–d). Compared with PBS, C9orf72-631 decreased the average number RNA foci per nucleus from 11 to 4 in motor neurons of the spinal cord (Fig. 4e, f). C9orf72-631 also significantly decreased the amount of DPR in spinal cord (68.7%, *P* < 0.0001, one-way ANOVA) and cortex (Fig. 4f, 64.9%, *P* = 0.0004, one-way ANOVA). C9orf72-296, the stereorandom oligonucleotide of identical sequence harboring industry-standard chemical modifications, decreased polyGP to a lesser extent in the spinal cord (24.4%, *P* = 0.0506, one-way ANOVA) and cortex (32%, *P* = 0.0484, one-way ANOVA). C9orf72-631 accumulated in the spinal cord (15.21 μg/g) and cortex (15.99 μg/g) of C9BAC mice more than either C9orf72-296 (0.83 and 8.75 μg/g, respectively) or NTC, which had already fallen below the limit of detection of the assay.

Taken together, these data indicate that C9orf72-631 distributes to the spinal cord and cortex, engages with *C9orf72* transcripts in C9BAC mice and leads to potent and preferential knockdown of V3 over other variants. Two 50 µg doses of C9orf72-631 impact molecular phenotypes associated with C9-ALS/FTD, including the accumulation of RNA foci and DPRs, at 2 weeks.

### C9orf72-631 sustainably impacts pathogenic signatures in C9BAC mice

**Fig. 5:**
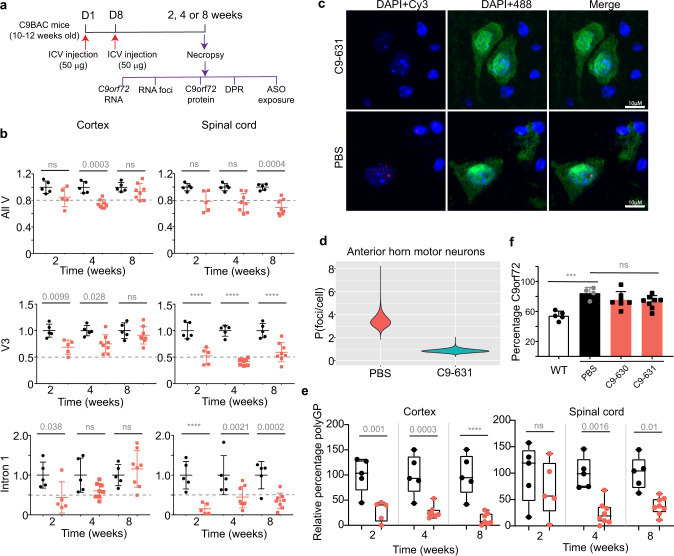
Stereopure oligonucleotides show durable activity in C9BAC mice. **a** Schematic representation of dosing regimen for 8-week study, with mice administered oligonucleotide on days (D) 1 and 8 and evaluated at 2, 4, or 8 weeks. **b** Relative transcript levels of all variants (top), V3 (middle), or intron 1-containing transcripts (bottom) in cortex (left) or spinal cord (right) at 2-, 4-, and 8-weeks post-dose are shown for C9orf72-631-treated (coral, *n* = 7) and PBS-treated mice (black, *n* = 5). *C9orf72* transcripts are standardized to *Hprt*, individual points indicate data from one mouse, and data are presented as mean ± SD. Two-way ANOVA with Sidak’s multiple comparisons. Exact *P* values are indicated, ns non-significant. **c** Representative images from evaluation of four mice per treatment group, showing spinal cords of mice treated with C9orf72-631 (top) or PBS (bottom) and stained for RNA foci (Cy3-labeled probe, red), nuclei (DAPI, blue), and motor neurons (anti-NeuN, green). 10 μm scale bars are shown. **d** Quantification of RNA foci per cell in anterior horn motor neurons in mice treated with PBS (fuschia) or C9orf72-631 (aqua) (Supplementary Fig. 8). **e** Relative percentage of DPR proteins detected in cortex (left) or spinal cord (right) for PBS (black, *n* = 5) or C9orf72-631 (coral, *n* = 7) at 2-, 4- and 8-weeks post-dose. Box plots show minima to maxima with horizontal lines depicting means. Two-way ANOVA with Sidak’s multiple comparisons. **f** Normalized percentage expression of C9orf72 protein in the spinal cord of wild-type (*n* = 5) or C9BAC mice treated with PBS (black, *n* = 4), C9orf-630 (coral, *n* = 7) or C9orf72-631 (coral, *n* = 8) at 8 weeks. Data are presented as mean ± SD. Two-way ANOVA with Dunnett’s multiple comparisons. ns, non-significant. Source data are provided as a Source Data file. ASO antisense oligonucleotide, ICV intracerebroventricular, DPR dipeptide-repeat protein; PBS phosphate-buffered saline, All V all variants, WT wild type mice, P probability; polyGP, polyglycine–proline.

In a longer study, two 50 μg doses of C9orf72-631 preferentially depleted V3 compared with all variants in the spinal cord for at least 8 weeks and in the cortex for at least 4 weeks (Fig. 5a, b). In the spinal cord, C9orf72-631 significantly decreased V3 (41–59%, *P* < 0.001, two-way ANOVA) and intron 1-containing transcripts (55–85%, *P* < 0.0001) at all time points, while having less impact on the expression of all variants (7–26%). In the cortex, C9orf72-631 decreased V3 (25–31%) at 2-week and 4-week time points. The magnitude of knockdown of all variants (15–25%) was less than that for V3 or intron 1-containing transcripts at all time points. Consistent with its impact on *C9orf72* transcripts, C9orf72-631 accumulated in and persisted in the spinal cord (5.23, 4.19, 2.66 μg/g at 2, 4, and 8 weeks, respectively) and cortex (8.69, 4.59, 5.92 μg/g at 2, 4, and 8 weeks, respectively). Similar results were obtained with the related oligonucleotide, C9orf72-630 (Supplementary Fig. 8). We visualized C9orf72-631 in spinal cord neurons at 8 weeks (Supplementary Fig. 8), confirming that it penetrates motor neurons in the CNS.

Importantly, the decreased pathological signatures for C9-FTD/ALS in response to C9orf72-631 that we detected at 2 weeks were largely sustained in the 8-week study. Sense RNA foci were decreased at the 95% confidence interval in the spinal cord compared with PBS-treated mice for up to 8 weeks (Fig. 5c, d). DPR proteins were significantly decreased in the spinal cord (60–85%, *P* < 0.01, two-way ANOVA) and cortex (52–91%, *P* < 0.01, two-way ANOVA, Fig. 5e) compared with PBS-treated mice. Importantly, C9orf72 protein expression in the cortex was not substantially different between C9orf72-631 and PBS-treated groups at 8 weeks (Fig. 5f). These data demonstrate that C9orf72-631 leads to durable knockdown of pathogenic *C9orf72* transcripts as well as a durable suppression of molecular signatures associated with C9-ALS/FTD in the cortex and spinal cord of C9BAC mice.

### C9orf72-631 protects motor neurons from glutamate toxicity

**Fig. 6:**
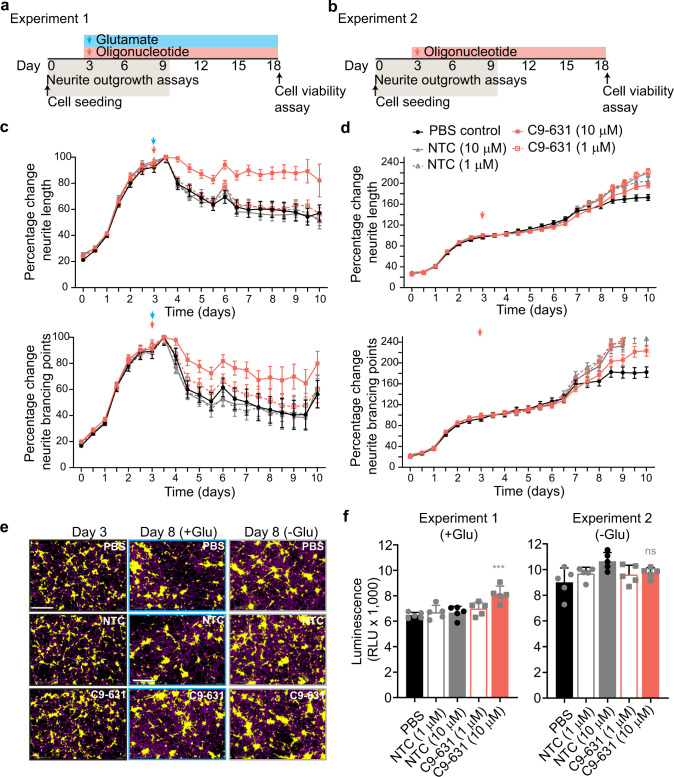
Stereopure oligonucleotide protects against glutamate toxicity in C9-ALS motor neurons. Schematic representation of experimental conditions in C9-ALS motor neuron assays for experiment 1 (**a**) and experiment 2 (**b**). **c** Percentage change in neurite length (top) and neurite branching points (bottom) over time (days) for C9-ALS motor neurons treated as shown in panel **a** with the indicated oligonucleotide. Data are presented as mean ± SEM, *n* = 3. **d** Percentage change in neurite length (top) and neurite branching points (bottom) over time (days) for C9-ALS motor neurons treated as shown in panel **b** and with the indicated oligonucleotide. Data are presented as mean ± SEM, *n* = 3. **e** Representative images of C9-ALS motor neurons pre-treatment (day 3) or post (day 8) in the presence or absence of glutamate (Glu), with PBS, 10 μM NTC or 10 μM C9orf72-631. Scale bar is 400 μm. Images were taken every 12 h for all samples (*n* = 3 per time point). This experiment was performed twice. **f** Relative survival (luminescence) for C9-ALS motor neurons. Data are mean ± SD, *n* = 3. One-way ANOVA with Dunnett’s multiple comparisons. ***P = 0.0002, ns non-significant. This experiment was performed twice. Source data are provided as a Source Data file. PBS phosphate-buffered saline, NTC non-targeting control, Glu glutamate.

Human motor neurons differentiated from C9-ALS/FTD-derived iPSCs exhibit glutamate toxicity^15,24^, and interventions that preserve the expression of normal C9orf72 protein^15^ or that decrease the production of DPRs^24^ in these cells can alleviate this toxicity. Because we have shown that C9or72-631 can both decrease the production of DPRs and preserve the expression of normal C9orf72 protein, we hypothesized that this oligonucleotide should also protect against glutamate-induced neurotoxicity in C9-ALS iPSC-derived motor neurons. To test this hypothesis, we evaluated the impact of C9orf72-631 on human ALS motor neuron cultures treated with glutamate. We recorded neurite outgrowth data at early time points (0–10 days) before axons start to form bundles, which can interfere with data collection. In the first evaluation, we co-administered oligonucleotide or control treatment (NTC or PBS) with glutamate (Fig. 6a, Experiment 1). In these cells, glutamate-induced neurite-outgrowth defects when applied at day 3. 10 μM C9orf72-631 preserved neurite length (*P* < 0.0001, two-way ANOVA) and neurite branching (*P* < 0.0001, two-way ANOVA) compared with PBS (Fig. 6c–f). These benefits were not observed in ALS motor neurons evaluated in the absence of glutamate (Fig. 6b–f, Experiment 2). These benefits were also not observed in wild-type motor neurons or in ALS-mutant motor neurons with M337V mutation in *TARDBP* (TAR DNA-binding protein 43, Supplementary Fig. 9). These data demonstrate that C9orf72-631 protects against neurodegenerative phenotypes associated with glutamate toxicity only in C9orf72-ALS motor neurons.

## Discussion

We report the discovery of an oligonucleotide-targeting sequence called SS1b at the exon 1b–intron 1 junction of *C9orf72* that unexpectedly yields preferential RNase H-mediated knockdown of V3 and intron-1-containing transcripts with stereopure oligonucleotides. Chemical modifications to the initial oligonucleotide proved necessary to obtain both potent and selective activity through this sequence. These modifications include duplex stabilizing 2′-MOE modifications in the 5′-wing, less stabilizing 2′-OMe modifications in the 3′-wing, and control over PS backbone stereochemistry^26^. Importantly, the potency and preferential activity we observed in cellular models under free-uptake conditions translated in vivo to a mouse model.

We hypothesize that stereopure oligonucleotides act selectively through SS1b because they are excluded from *C9orf72* variants that interact with the splicing machinery, and we provide multiple lines of evidence supporting this mechanism. Oligonucleotides that target sequences near SS1b, which are not predicted to interact with the splicing machinery, do not produce preferential activity. An oligonucleotide that binds with high affinity to SS1b but that does not promote RNase H-mediated degradation disrupts splicing, increasing the prevalence of transcripts that retain intron 1. This finding supports the premise that the spliceosome is acting through SS1b on some transcripts. In biochemical experiments, pre-annealing a low-affinity, U1-RNA mimic to a *C9orf72*-RNA surrogate protects the surrogate from RNase H-mediated degradation even though this mimic is only partially complementary to the *C9orf72* RNA. This finding supports the idea that components of the spliceosome (e.g., U1 snRNP) may limit access to SS1b. Together, these experiments support our hypothesis that C9orf72-631 cannot access V2 due to the presence of splicing machinery at the exon 1b–intron 1 boundary.

We initially considered that stability differences among pre-mRNAs could drive selective activity towards the expansion-containing V3 pre-mRNA, as the intronic region is retained or stabilized by the G_4_C_2_-repeat expansion^5,31^. However, if stability differences resulting from the expansion was the main driver for selectivity, our oligonucleotides should maximally deplete 50% of V3 (affecting only those derived from the expanded allele). Because we observe 90% knockdown of V3, we believe C9orf72-631 is promoting degradation of both V3 variants. Thus, pre-mRNA stability differences may contribute to C9orf72-631’s preferential activity but they are unlikely the only driver.

To our knowledge, this is the first report of oligonucleotides that yield preferential activity against alternatively spliced transcripts based on differences in the protection of specific variants by a protein complex such as the spliceosome. Nusinersen, an FDA-approved exon-skipping oligonucleotide, targets the intronic splicing silencer N1 (ISS-N1) in *SMN2*^36^; however, oligonucleotides including nusinersen that target ISS-N1 do not promote RNase H-mediated degradation of the target RNA. They are believed to act by disrupting an inhibitory structure that represses splicing, thereby enabling splicing and protein expression^36^. Similarly, exon-skipping phosphorodiamidate morpholino oligomers (PMOs) and oligonucleotides for Duchenne muscular dystrophy are believed to block the spliceosome access to sequences necessary for splicing^37^. By contrast, C9orf72-631 is selective because it does not disrupt the splicing machinery in V2. The preferential activity we observe at SS1b suggests that comparable targeting sites may be available in other disease-associated genes. It may be possible to design oligonucleotides that selectively target pathogenic RNAs derived from any gene that encodes multiple transcripts if those transcripts engage in different interactions with proteins.

C9orf72-631 preferentially decreases expression of pathogenic transcripts. It decreases the expression of V3 more than all variants, providing evidence that the oligonucleotide is most active against variants that initiate upstream of the repeat expansion. C9orf72-631 also has a more profound impact on intron-1-containing variants than on V3. The intron 1 primers detect more pathological variants than the V3 primers, including mis-spliced variants of V1 and V3, stabilized transcripts containing the expansion, and antisense transcripts. The increased potency of C9orf72-631 for intron 1-containing variants over V3 provides additional support for C9orf72-631’s preferential activity against pathogenic variants.

C9orf72-631 led to sufficiently potent and selective knockdown of pathogenic *C9orf72* variants to disrupt gain-of-toxicity mechanisms associated with pathogenic RNA expression, including the accumulation of RNA foci and DPRs for up to 8 weeks in C9BAC mice. Antisense transcripts are also produced from the expansion-containing allele^5,8,10^. Although, our oligonucleotides were designed to target sense transcripts, we believe they may also address antisense transcripts. Importantly, they do not increase the expression of antisense transcripts, which has been highlighted as a potential complication for a sense-targeting approach^10,38^. Our primers for intron 1 correspond to the *C9orf72* region that produces antisense transcripts. In the qPCR assay, which is not strand-specific, we observe >70% decrease in these transcripts in the spinal cord. The magnitude of this response indicates that C9orf72-631 does not lead to an increase in the expression of antisense transcripts and suggests that it may deplete both sense and antisense transcripts. Similarly, polyGP DPRs can be encoded by sense and antisense transcripts. Because C9orf72-631 potently and durably decreases polyGP DPRs, it is unlikely that antisense transcripts are spared.

After treatment with C9orf72-631, C9orf72 protein expression is maintained. No prior studies using oligonucleotides to target *C9orf72* have reported whether they impacted C9orf72 protein expression^8,11,30^. Multiple reports^15,16,24^ indicate that haploinsufficiency for C9orf72 can contribute to C9-ALS/FTD, so this is an important outcome. C9orf72-631 also protects ALS motor neurons from phenotypes associated with glutamate-induced neurotoxicity, providing further evidence that it addresses the major pathogenic signatures associated with the repeat expansion.

With C9orf72-630 and C9orf72-631, we achieve potent, selective and durable knockdown of *C9orf72* pathogenic variants in mice at lower doses than for previously reported oligonucleotides (100 µg total dose herein compared with 350^11^ and 500 µg^8^). C9orf72-631 exhibited CNS tissue half-lives longer than 1 month, which is consistent with the durability of its effects. Thus, stereopure oligonucleotides targeting SS1b represent a modality with the potential to address C9-ALS/FTD.

## Methods

### Oligonucleotides

We obtained PO-based and stereorandom PS-modified oligonucleotides from Integrated DNA Technologies or by using a standard solid-phase oligonucleotide synthesis protocol. We synthesized and purified chemically modified, stereopure oligonucleotides as described with minor modifications^24,36,37^. We characterized stereopure oligonucleotides by LC–HRMS and HPLC (Supplementary Fig. 10). Sequence and chemistry of all oligonucleotides used in this study are shown in Supplementary Table 1.

### Reversed-phase HPLC

10 µL of a 5 µM solution of each oligomer was injected onto an analytical HPLC column (Poroshell 120 EC—C18, 2.7 µm, 2.1 × 50 mm, Agilent) using Buffer A (200 mM hexafluoroisopropanol and 8 mM triethylamine in water) and Buffer B (methanol) as eluents with a gradient of Buffer B from 5% to 30% at 60 °C. UV absorbance was recorded at 254 and 280 nm.

### DNA constructs

For luciferase reporter assays, we introduced *C9orf72* sequences into the NotI site of the psiCHECK-2 vector (Promega), which is in the middle of the 3′-UTR of the hRluc gene. *C9orf72* sequences encompassed ~1 kb of DNA surrounding intron 1, including exons 1a and 1b and downstream regions of the gene.

### Animals

All animal experiments were performed at Biomere Biomedical Research Models (Worcester, MA) in compliance with Biomere’s Institutional Animal Care and Use Committee guidelines for care and use of animals. Mice were on a 12-h light–dark cycle. Food (lab diet 5001) and water were available ad libitum. Housing rooms were maintained at 20–26 °C and relative humidity was 30–70%. For in vivo studies, we used C9BAC transgenic mice^17^, Tg(C9orf72_3) No. 023099, Jackson Laboratories), which have several tandem copies of the *C9orf72* transgene, with each copy having between 100 and 1000 repeats. For studies herein, we selected mice expressing ≥500 repeats that were 10–12 weeks old. We utilized both male and female mice. For intracerebroventricular cannulation under stereotaxic surgery, we anesthetized mice (avertin) and placed them on a rodent stereotaxic apparatus; they were then implanted with a stainless-steel guide cannula in one of their lateral ventricles (coordinates: −0.3 mm posterior, +1.0 mm lateral, and −2.2 mm vertically from bregma), which we secured in place using dental cement. Mice were allowed a 1-week recovery period.

In the dose-escalation study, we administered 8, 20, or 50 µg of ASO in 2.5 µL on days 1 and 8, and mice were necropsied 2 weeks after the first injection. For the 2-week multi-dose study (Fig. 4), we administered 50 µg oligonucleotide in 2.5 µL on days 1 and 8, and mice were necropsied as above. For the duration of action study (Fig. 5, Supplementary Fig. 8), we assessed mice at three time points (2, 4, and 8 weeks; *n* = 5–8 per group per time point) after dosing. For the single-dose duration study (Supplementary Fig. 6), we injected 100 μg oligonucleotide in 2.5 µL on day 1 and assessed mice 48 h (*n* = 6 per group), 1 week (*n* = 6), 2 weeks (*n* = 6), 8 weeks (*n* = 6), and 12 weeks (*n* = 6) after dosing. At necropsy, mice were transcardially perfused with saline under avertin anesthesia. We rapidly removed brains from the skull; we processed one hemisphere for histological analyses (drop-fixed in 10% formalin) and the other, we dissected into cortex, hippocampus, striatum, and cerebellum, which were frozen on dry ice for biochemical analyses. Similarly, we dissected spinal cord and froze it on dry ice or processed it for histological analyses.

### Cellular models

#### Cos-7 cells

We obtained Cos-7 cells from ATCC.

#### iPSCs

iPSCs derived from patient fibroblasts came from a chromosome 9 open-reading frame 72 (C9orf72)-associated female patient with ALS (64 years old, RUCDR Infinite Biologics). iPSCs were maintained as colonies on Corning Matrigel matrix (Millipore Sigma) in mTeSR1 medium (STEMCELL Technologies).

#### iPSC-derived neurons and ALS cortical neurons

Neural progenitors were produced by using STEMdiff Neural System (STEMCELL Technologies). iPSCs were suspended in an AggreWell800 plate and allowed to grow as embryoid bodies in STEMdiff Neural Induction Medium for 5 days, with daily 75% medium changes. Embryoid bodies were harvested with a 37 μm cell strainer and plated onto Matrigel-coated plates in STEMdiff Neural Induction Medium, which was changed daily for 7 days, with 85–95% of embryoid bodies exhibiting neural rosettes 2-days post-plating. Rosettes were manually selected and transferred to plates coated with poly-l-ornithine and laminin in STEMdiff Neural Induction Medium (STEMCELL Technologies). The medium was changed daily until cells reached 90% confluence (7 days) and considered to be neural progenitor cells (NPCs). NPCs were disassociated with TrypLE (ThermoFisher) and passaged at a ratio of 1:2 or 1:3 on poly-l-ornithine/laminin plates in a neural maintenance medium (NMM; 70% DMEM, 30% Ham’s F12, 1X B27 supplement) supplemented with growth factors (20 ng/mL FGF2, 20 ng/mL EGF, 5 µg/mL heparin). For maturation into neurons, NPCs were maintained and expanded for <5 passages, and at >90% confluence were passaged 1:4 onto poly-l-orinithine/laminin-coated plates in NMM supplemented with growth factors. The next day, Day 0 of differentiation, the medium was changed to fresh NMM without growth factors. Differentiating neurons were maintained in NMM for ≥4 weeks, with twice weekly 50% medium changes. Cortical neurons were replated with TrypLE at a density of 125,000 cells/cm^2^ as needed.

#### iPSC-derived ALS motor neuron differentiation

Motor neurons derived from the same patient iPSC line were differentiated by BrainXell and seeded with their standard protocol. Spinal motor neurons were generated from a human ALS iPSC line harboring a *C9ORF72* mutation (Target ALS ID: TALS9-9.3; Rutgers ID: 150000002; NINDS ID: ND50000), a human healthy iPSC line (400238-1M, iXCell), and a human ALS iPSC line harboring a heterozygous *TARDB* mutation, encoding TDP-43 M337V (400311-MN-TDP43HETM337V-CR8-C1, iXCells). Directed differentiation was performed as described^39^. Briefly, iPSCs were treated with small molecules CHIR99021, DMH-1, and SB431542 for 6 days to induce SOX1^+^ neuroepithelial progenitors (NEPs). The NEPs were split and treated with CHIR99021, DMH-1, SB431542, retinoic acid, and purmorphamine for another 6 days to generate OLIG2^+^ motor neuron progenitors (MNPs). These OLIG2 + MNPs were expanded and frozen at the point of early differentiation to motor neurons.

#### Primary human fibroblasts from human ALS patients

*C9orf72*-repeat expansion containing fibroblasts were obtained at UMass Medical School under the auspices of an IRB-approved protocol for skin biopsy and fibroblast culture (IRB No. 13019). C9-2. RB19842 were obtained from a 64-year old, Caucasian female; C9-3. RB19895 were obtained from a 59-year-old Caucasian male. Both subjects had confirmed *C9orf72*-repeat expansions and met diagnostic criteria for ALS. Specimens were obtained from live subjects via dermal punches to the forearm, with no pathology noted at the time of collection, and were cut into small pieces and placed directly in a culture dish with DMEM supplemented with 15% fetal bovine serum. Once established, fibroblasts cultures were expanded and subsequently aliquoted and frozen at low passage number, using standard protocols (thermofisher.com/cellculturebasics).

#### Primary C9BAC cortical neurons

Primary cortical neurons were generated from E15.5 C9BAC transgenic mouse embryos^17^. Embryos were removed at E15.5 from pregnant wild-type C57BL/6 females crossed with homozygous C9BAC males. Cortical tissue of each embryo was dissected on ice-cold Hank’s balanced salt solution (ThermoScientific). Pooled tissue was minced and digested with 0.05% trypsin–EDTA (Life Technology) at 37 °C for 12 min. Digestion was halted by addition of 10% FBS/DMEM. Cells were triturated, resuspended in neurobasal media supplemented with Glutamax (ThermoScientific), 2% penicillin/streptomyocin and B27 supplement (ThermoScientific) and seeded at 0.5 × 10^6^ cells/well in six-well plates pre-coated with poly-ornithine (Sigma).

### Southern blot

Genomic DNA was isolated from ALS iPSCs, ALS motor neurons and C9BAC transgenic mice using Gentra Puregene Tissue kits (Qiagen). 10 μg DNA was digested overnight with AluI and DdeI at 37 °C and then separated by electrophoresis on a 0.6% agarose gel, transferred to positively charged nylon membrane (Roche Applied Science), cross-linked by exposure to UV light, and hybridized overnight at 55 °C with digoxigenin-labeled (G_2_C_4_)_5_ DNA probe in hybridization buffer (EasyHyb, Roche). The probe was detected using 150 mU/mL anti-digoxigenin antibody (Catalog no. 11093274910, Roche) and CDP-Star reagent as recommended by the manufacturer.

### Thermal denaturation (*T*_m_)

Equimolar amounts of surrogate RNA (5′-GGUGGCGAGUGGGUGAGUGAGGAG), U1 mimic (5′-AUACUUACCUGG) and/or oligonucleotide were dissolved in 1× PBS to obtain a final concentration of 1 μM of each strand. Duplex samples were then annealed by heating at 90 °C, followed by slow cooling to 4 °C and storage at 4 °C. UV absorbance at 254 nm was recorded at intervals of 30 s as the temperature was raised from 5 or 15 °C to 95 °C at a rate of +0.5 °C per min, using a Cary Series UV–Vis spectrophotometer (Agilent Technologies). Absorbance was plotted against the temperature and the *T*_m_ values were calculated by taking the first derivative of each curve.

### RNase H assays

For RNase H assays, we incubated heteroduplexes with human RNase HC (prepared as described^26^) at 37 °C. We prepared duplexes by mixing equimolar solutions of oligonucleotide and/or U1 mimic and RNA yielding a final concentration of 20 µM. Each reaction contained 5 µM ASO-RNA, U1 mimic-RNA, or ASO–U1 mimic–RNA heterocomplexes in RNase H buffer (75 mM KCl, 50 mM Tris–HCl, 3 mM MgCl_2_, 10 mM dithiothreitol, pH = 8.3) in a reaction volume of 50 µL. The pre-mixtures were incubated at 37 °C for 10 min prior to the addition of enzyme + U1 mimic, enzyme + ASO, or enzyme alone with final concentration ratios 2000:1, 1000:1, or 500:1 substrate:RNase HC. We quenched the reactions at 5, 10, 15, 30, 45, and 60 min using 7.0 µL of 250 mM EDTA disodium solution in water. For the 0 min-time point, we added EDTA to the reaction mixture before enzyme. We recorded UV absorbance at 254, 210, and 280 nm for each reaction after injection onto an Agilent Poroshell 120 EC-C18 column (2.7 µm, 2.1 × 50 mm) at 60 °C using a gradient of Buffer A (200 mM HFIP and 8 mM triethylamine) and Buffer B (methanol). We integrated the peak areas from the chromatograms, corresponding to full-length RNA oligomer, normalized them compared to the antisense strand. We plotted the percentage RNA remaining, with the 0 min-time point defined as 100%, to show relative rates of RNA cleavage (*n* = 3). We analyzed the data with two-way ANOVA. Error bars indicate SEM.

### Duplex analysis for RNase H assays

We mixed equimolar solutions of ASO, RNA, and/or U1 to prepare duplexes at a final concentration of 20 µM. We prepared three complexes: ASO + RNA, RNA + U1, and ASO + RNA + U1. The mixtures were heated to 90 °C for 2 min and allowed to cool slowly to room temperature for more than 4 h. The D1000 ladder and sample buffer (7 mM KCl, 20 mL phosphate buffer, 20 mM guanidine–HCl, 80 mM NaCl, 20 mM acetate) were equilibrated at room temperature for 30 min. Samples for analysis were prepared by mixing 1:1 with D1000 sample buffer. The samples and ladder were mixed thoroughly using the IKA vortex at 500 × *g* for 1 min. Samples were centrifuged to ensure the full volume settled to the bottom of the tube. We analyzed duplexes on 4200 Agilent Tape station (software A.02.02) using high sensitivity D1000 screentape (sizing range 35–1000 bp) according to manufacturer’s protocol.

### Luciferase screening assay

We generated a luciferase construct containing sequences from the human C9orf72 gene (158–900 base pairs) in the 3′-UTR of the renilla luciferase gene in psiCHECK2 vector. An ASO targeting this sequence should decrease renilla luciferase signal without affecting the firefly luciferase signal. We normalized renilla to firefly luciferase signals to compare the relative activity of ASOs versus a non-targeting control (NTC). We delivered ASOs (15 or 30 nM) and the luciferase reporter constructs (20 ng) by transfection with Lipofectamine 2000 into Cos-7 cells. The firefly and renilla luciferase signals were quantified with a plate reader (Molecular Devices Spectramax M5 with SoftMax pro 7.0 software) 48-h post-transfection. We performed three biological replicates per experiment.

### Half-maximal inhibitory concentration (IC_50_)

We calculated IC_50_s in full dose–response assays (10, 2.5, 0.62, 0.16, 0.04, and 0.001 µM) in ALS motor neurons. Briefly, we delivered oligonucleotides gymnotically and evaluated transcript levels as described above after one week. We fit data using non-linear regression for variable slope (four parameters) using GraphPad software.

### ASO delivery to cellular models

Human ALS cortical neurons were maintained in NMM for at least 4 weeks in 24-well plates (250,000 cells per well) and treated with 1 µM of the indicated ASO gymnotically (with no transfection reagent) for one week. Primary neurons from C9BAC transgenic mice were treated with ASO gymnotically at the indicated dose 5 days after culture and collected 15 days after treatment. Human ALS motor neurons were seeded in 12-well plates (280,000 cells per well) from frozen stocks and treated gymnotically on day 7 and harvested on day 14. 50 μL of a growth factor cocktail containing 10 ng BDNF, 10 ng of GDNF, and 1 ng of TGF-β1 were added on day 10 without changing the medium. C9-patient-derived fibroblasts were plated in 10 cm dishes, and the ASOs were transfected using Lipofectamine RNAiMax Reagent (ThermoScientific). The cells were harvested 72 h after treatment.

### C9orf72 transcript quantification assays

In human ALS cortical neurons, ALS motor neurons and transgenic mouse tissues, total RNA was extracted using Trizol (Invitrogen) according to manufacturer’s protocol. For each sample, total RNA was eluted in 29.5 μL of RNase-free water followed by the addition of 2 μL (4U) of DNase I (New England Biolabs, M0303L) and 3.5 μL of 10× reaction buffer. Samples were incubated at 37 °C for 15 min for gDNA removal. EDTA was added to 5 mM final concentration, and DNase I was heat inactivated at 75 °C for 10 min. RNA was reverse transcribed with High-Capacity RNA-to-cDNA™ Kit (Applied Biosystems) according to manufacturer instructions. Transcripts were quantified using Taqman assays. We used the following Taqman probes: Hs00376619_m1 (FAM) (Catalog # 4351368, ThermoFisher) for C9orf72 All Transcripts (common on V1–V3); Hs00948764_m1(FAM) (Catalog # 4351368, ThermoFisher) for C9orf72 V3 transcripts; Hs02800695_m1 for human *HPRT1* transcripts (Catalog# 4448486, ThermoFisher). *C9orf72* intron 1 and Mouse *Hprt1* Taqman assays were designed in house (see Supplementary Table 2). qPCR reaction: 3 min at 95 °C, 40 cycles of 10 s at 95 °C, and 30 s at 60 °C. In C9-patient-derived fibroblasts and C9BAC primary cell lines, total RNA was isolated using Trizol (ThermoScientific) and subsequently treated with DNase I (Qiagen). One µg of total RNA was reverse transcribed into cDNA using random hexamers and MultiScribe reverse transcriptase (ThermoScientific) following the manufacturer’s instructions. Quantitative PCR was performed on a StepOnePlus Real-Time PCR (qRT-PCR) system using SYBR Green Master Mix (Applied Biosystems) and 0.2 μM of forward and reverse primers as described^29^. Ct values for each sample and transcript were normalized to *Hprt*. The 2exp (−ΔΔCt) method was used to determine the relative expression of each transcript using Bio-Rad CFX manager 3.1 with BioRad CFX Maestro 1.1 version 4.1.2433.1219 software.

### Tissue processing for transcript analyses by PCR and ASO quantification by hybridization ELISA

We dissected and fresh-froze tissues in pre-weighed Eppendorf tubes. We calculated tissue weight by re-weighing. For lysis, we added four volumes of Trizol or lysis buffer (4 M guanidine; 0.33% N-lauryl sarcosine; 25 mM sodium citrate; 10 mM DTT) to 1-unit weight (4 μL of buffer for 1 mg tissue) and homogenized tissue at 4 °C using precellys until all the tissue pieces were dissolved. 30–50 μL of tissue lysates were saved in 96-well plates for pharmacokinetic (PK) measurement. The remaining lysates were either stored at −80 °C (when in lysis buffer) or used for RNA extraction (when in Trizol).

We utilized the following probes to selectively quantify the ASOs used in this study by hybridization ELISA: Capture probe: “C9-Intron-Cap” /5AmMC12/TGGCGAGTGG; Detection probe: “C9-Intron-Det”: GTGAGTGAGG/3BioTEG/. We coated maleic anhydride-activated 96-well plate (Pierce 15110) with 50 μL of capture probe at 500 nM in 2.5% NaHCO_3_ (Gibco, 25080-094) for 2 h at 37 °C. The plate was then washed three times with PBST (PBS + 0.1% Tween-20), blocked with 5% fat free milk-PBST at 37 °C for 1 h. Payload ASO was serially diluted into matrix. This standard together with original samples were diluted with lysis buffer (4 M guanidine; 0.33% N-lauryl sarcosine; 25 mM sodium citrate; 10 mM DTT) so that the ASO amount in all samples was <50 ng/mL. 20 μL of diluted samples were mixed with 180 μL of 333 nM detection probe diluted in PBST, then denatured (65 °C, 10 min, 95 °C, 15 min, 4 °C∞). 50 μL of the denatured samples were distributed in blocked ELISA plates in triplicates, and incubated overnight at 4 °C. After three washes with PBST, 50 μL of 1:2000 streptavidin-AP (SouthernBiotech, 7100-04) in PBST was added, 50 μL per well and incubated at room temperature for 1 h. After extensive washes with PBST, 100 μL of AttoPhos (Promega S1000) was added, incubated at room temperature in the dark for 10 min and read on the plate reader (Molecular Device, M5) fluorescence channel: Ex435 nm, Em555 nm. The ASO in samples were calculated according to standard curve by four-parameter regression. The lower limit of detection was 1.25 μg ASO per gram of tissue.

### Stability in mouse brain homogenate

We determined the stability of the ASOs in mouse brain homogenate by adding 5 µL of each oligo solution (200 µM) to 45 µL of mouse brain homogenate (prepared in-house, 20 mg/mL). We incubated each reaction at 37 °C while shaking at 400 rpm. We used a 20mer DNA sequence as a positive control to assess the performance of the assay. Because it does not incorporate chemical modifications to protect against nuclease degradation, DNA degrades rapidly. We terminated reactions, which we performed in triplicate, at each time point (0–5 days) by adding 50 µL of Stop buffer (2.5% IGEPAL, 0.5 M NaCl, 10 mM EDTA, 50 mM Tris, pH = 8.0) followed by vortexing. We than added 20 µL of internal standard (50 µM: 5′-GCGTTTGCTCTTCTTCUUGCGTTTTUU-3′), 250 µL of 2% ammonium hydroxide and 100 µL of phenol:chloroform:isoamyl alcohol (25:24:1) to each tube. After vortexing, we spun each reaction at 35,000x*g* at room temperature for 30 min and repeated the above extraction protocol with the aqueous layer using 150 µL of chloroform. After transferring the new aqueous layer to a new tube, we dried and then reconstituted each sample with water in a volume of 100 µL. 2 µL of the mixture was injected to Q Exactive mass spectrometer (Thermo Fisher Scientific) using Agilent Poroshell column (120, EC-C18 2.7 µm, 2.1 × 50 mm) and mobile Phase A (400 mM HFIP, 15 mM TEA in water) and Mobile Phase B (Methanol). We used Xcalibur TM (version 4.0.27.10, Thermo Fisher Scientific) for data capture and to calculate peak areas and peak area ratios of analytes to the internal standard. Reduction in analyte amount was used to evaluate the extent of in vitro stability. We calculated mean and standard deviation from three technical replicates.

### Fluorescence in situ hybridization (FISH) detection of RNA foci

We used a 5′-end, Cy3-conjugated (G_2_C_4_)_3–4_ probe to detect sense-repeat expansions and a Cy3-conjugated (G_4_C_2_)_3_ probe to detect antisense repeat expansions (probes from Integrated DNA Technologies). 10 μm frozen sections were cut on the cryostat. Frozen sections were fixed in 4% paraformaldehyde in PBS for 20 min and then incubated in cold 70% ethanol for at least 30 min at 4 °C. Following rehydration in (40% formamide, 2× SSC), the slides were blocked with hybridization solution (40% formamide, 2× SSC, 20 μg/mL BSA, 100 mg/mL dextran sulfate, and 250 μg/mL yeast tRNA, 2 mM vanadyl sulfate ribonucleosides) for 30 min at 55 °C and then incubated with 200 ng/mL of denatured probe in hybridization solution at 55 °C for 3 h. The slides were washed three times with 40% formamide in 2× SSC and once with PBS. To co-stain neurons, the sections were permeabilized in 0.5% Triton X-100 in PBS for 15 min on ice and blocked in 2% normal goat serum in PBS for 1 h. The sections were then incubated overnight at 4 °C with NeuN antibody (1:500 dilution, anti-NeuN antibody, MAB377, Millipore). The sections were then washed three times in PBS and incubated for 1 h at room temperature with goat anti-mouse secondary antibody with Alexa Fluor 488 (1:500 dilution, Thermo Fisher, Cat. no. A3273). Sections were washed and autofluorescence of lipofuscin was quenched by 0.25% of Sudan Black B in 70% ethanol. Sections were then mounted in Prolong Gold Antifade reagent with DAPI (ThermoFisher). Confocal images of 488 nM, Cy3 and DAPI channels were taken with a RPI spinning disk confocal microscope (Zeiss) at ×40 magnification using image capture software MetaMorph version 7.10.3.279 (Molecular Devices). The stacked images were merged using Z Project function.

### Quantification of RNA foci

After detection with FISH (described above), nuclei are identified with the mask, and the area of each nucleus is measured. The green channel is stained with NeuN as a marker of neurons. Based on the observation that NeuN stains bigger nuclei in anterior horn region, cells with nuclei bigger than 78 μm^2^ are identified as motor neurons for high-throughput foci counting. The Cy3 channel was used to identify foci, find maximum function was used to identify single points with a set noise tolerance (30–90, set for each experiment, constant between samples). Within each nucleus, the integrated density was recorded and divided by 255 as the number of foci in this nucleus. A probabilistic model was used to calculate the posterior of foci/cell; foci count and cell count were modeled with the Poisson distribution using the function *rpois* in the R Stats package. Posterior samples were obtained using a Monte Carlo method. Inference was performed on the posteriors by subtracting the PBS (i.e., control) posterior from the posterior for each treatment, including itself. If the 95% highest posterior density for the compound treatments did not cover zero, then these treatments were considered credibly different from PBS at the 95% confidence level.

### PolyGP quantification

Brain and spinal cord samples were homogenized in four volumes RIPA (50 mM Tris, 150 mM NaCl, 0.5% DOC, 1% NP40, 0.1% SDS and Complete protease inhibitor, pH 8.0) by shaking in a Precellys instrument with 1.4 mm zirconium oxide beads. Samples were centrifuged at 12,100x*g* for 10 min at 4 °C, and total protein concentration of clarified lysate was determined with 600 nm Protein Assay Reagent (Pierce). MSD Small-Spot plates were coated with 1 μL of a 10 μg/mL solution of a polyclonal capture antibody (rabbit anti-polyGP; AB1358, Millipore) and incubated at 4 °C overnight. The next day, plates were washed with PBST, blocked with a 10% FBS/PBST solution for 1 h at room temperature, and then washed with PBST and incubated with 50–120 μg of brain lysate (diluted 1:4 or 1:5 into 10% FBS/PBST) for 2–4 h. Plates were washed with PBST and incubated with Sulfo-tag-conjugated detection antibody (rabbit anti-polyGP; AB1358, Millipore) for 1 h at room temperature. Plates were washed with PBST and incubated with 150 μL of MSD Read Buffer T 1× and read in an MSD QuickPlex SQ 120 plate reader with Discovery Workbench 4.0.12 (LSR_4_0_12) software. A standard curve of recombinant purified polyGPx30 was prepared in a matrix of wild-type mouse cortex or spinal cord homogenate. After subtracting the background signal measured from empty-wells, a linear best-fit regression line for the standard curve was used to interpolate the concentration of polyGP per microgram of tissue. Data are reported as relative percentage of polyGP, reflecting percentage of polyGP detected compared with PBS-treated samples.

### Western blots

We quantified the expression of C9orf72 protein by western blotting. Briefly, proteins from RIPA extracts were size fractionated with precast 4–12% SDS–PAGE (Criterion gels, Bio-Rad) and transferred onto PVDF membrane. To detect C9orf72, we used the mouse monoclonal anti-C9orf72 antibody GT779 (1:2000, GeneTex Inc, Cat. no. GTX632041) and 1:1000 dilution of the secondary DyLight 594 goat anti-rabbit antibody (Thermo Fisher, Cat. no. 35560). We visualized and quantified blots using the Odyssey imaging system (LI-COR Biosciences Image studio V5.2). Full-sized blots are shown (Source Data file).

### PK analysis

The mean tissue concentration–time profiles of C9orf72-631 were modeled using a one-compartment model with a first-order absorption rate and a first-order elimination rate. The tissue concentration was described by

1 $${C}_{\mathrm{t}} = {\mathrm{Dose}} * {{K_{\mathrm{a}}}/V} * \left( {{K_{\mathrm{{a}}}} - {K_{\mathrm{{e}}}}} \right) * ({\mathrm{exp}}\left( {{-K_{\mathrm{{a}}}} * {t}} \right){\mathrm{-exp}}\left( {{-K_{\mathrm{{e}}}} * {t}} \right))$$

where *C*_t_ represents the tissue concentration, dose represents administered amount, *K*_a_ represents absorption rate, *V* represents volume distribution, *K*_e_ represents elimination rate, and *t* represents time post-dose. The terminal half-life in tissues was derived as ln_2_/*K*_e_. A two-compartment model was also tested but appeared to be over-parameterized. All below the limit of quantification values were set to zero for the analysis. The model parameters were estimated using Phoenix^®^ WinNonlin^®^ 8.1 software program (Certara, Princeton, NJ, USA).

### ViewRNA in situ hybridization (ISH) assay

We adapted ViewRNA ISH Tissue 1-Plex Assay (ThermoFisher Scientific, Cat# QVT0051) for detection of ASOs in situ. Briefly, spinal cord biopsies were fixed in 10% neutral buffered formalin overnight at 2–8 °C, processed and embedded in paraffin. Paraffin sections (5 µm) were prepared and stored at room temperature until use. After baking slides at 60 °C for at least 1 h, we dewaxed in xylene (VWR Chemicals) for 10 min and rinsed in 100% ethanol (ThermoFisher Scientific). After air drying slides for at least 30 min at room temperature, we created a hydrophobic barrier before continuing with ViewRNA ISH standard protocol. We treated rehydrated slides with pre-heated target retrieval reagent for 10 min at 95 °C, followed by protease digestion (Protease QF 1:100 in 1× PBS, pre-warmed) at 37 °C for 15 min. We rinsed slides in 1× PBS with agitation and then treated them with QuantiGene ViewRNA miRNA probe sets for C9orf72-631, PPIB (positive control), and/or dapB (negative control) (ThermoFisher Scientific) diluted to 12.5 nM in pre-warmed Probe Set Diluent QT (300 µL per section) for 2 h at 40 °C. We stored rinsed slides at room temperature for up to 24 h. For signal amplification and detection, we incubated slides in working PreAmp1 QF solution diluted at 1:100 in prewarmed Amplifier Diluent QF for 30 min at 40 °C; we rinsed in wash buffer with agitation, which was followed by incubation in working Amp1 QF solution (1:100) in prewarmed Amplifier Diluent QF for 20 min at 40 °C. After rinsing, we incubated the slides in Label Probe-AP working solution (1:1000 in Label Probe Diluent QF) for 20 min at 40 °C and rinsed in wash buffer with agitation. We added AP-Enhancer Solution and incubated for 5 min at room temperature before adding Fast Red Substrate and incubating a further 30 min at 40 °C to develop red color deposit. Afterwards, we counterstained DNA with hematoxylin and/or Hoechst 33342 dye. The slides were mounted with ProLong Gold Antifade mounting medium (Molecular Probes, # P36930), and covered with a thin glass coverslip. For each spinal cord cross-section, the representative digital images were generated using a Zeiss Axio Observer microscope (Zeiss, Thornwood, NY, USA) under brightfield or fluorescent field using Zen 2.3 software.

### Neurite outgrowth and survival assays

Assays were performed in C9-ALS iPSC motor neurons (Lot no. 180818-0100A; BrainXell, Madison, WI), healthy iPCS motor neurons (Lot no. 400238-1 M, iXcells Biotechnologies, San Diego, CA), or TDP43 M337V iPSC motor neurons (Lot no. 400311-MN-TDP43HETM337V-CR8-C1, iXcells Biotechnologies, San Diego, CA) and were performed by iXCell Technologies (San Diego, CA). Briefly, motor neurons were seeded in 96-well plates (15,000 cells per well) in triplicate (Day 0). In experiment 1, neurons were treated with 10 µM glutamate and PBS or oligonucleotide (final concentration (*C*_f_ of 1 or 10 μM) on day 3. In experiment 2, neurons were treated with PBS or oligonucleotide (*C*_f_ of 1 or 10 μM) on day 3. For all experiments, a one-third media change was performed every 3–4 days. PBS, oligonucleotide, and/or glutamate, was replenished in the new media. The neurite length and neurite branch points were analyzed twice daily (every 12 h) for 10 days with Incucyte imaging system for live-cell analysis. Neurite length per cell-body cluster area and neurite branch points per cell-body cluster area were analyzed using IncuCyte S3 2017A (Essen BioScience, Ann Arbor, MI). Excitotoxicity was assessed in ATP-dependent cell viability assays with Cell TiterGlo (Promega, Madison, WI) at day 19. Survival was quantified using CellTiter-Glo Luminescent Cell Viability assay (Promega, Madison, WI) using SYNERGY HTX plate reader with Gen 5 3.09 software following manufacturer’s protocol.

### Statistical analyses

Unless otherwise indicated, data were analyzed by a one-way or two-way analysis of variance (ANOVA) followed by post-hoc analyses for multiple comparisons with appropriate test using GraphPad Prism software.

### Reporting summary

Further information on research design is available in the Nature Research Reporting Summary linked to this article.

## Supplementary information

Supplementary Information

Reporting Summary

Source data file

## Data Availability

The source data underlying Figs. 2b–d, 3b–d, 4b, c, e, f, 5b, d–f, 6c, d, and f, and Supplementary Figs. 1c, d, f–h, 2a–e, 3b–h, 6a, e, 7a, d, 8a, b, 9a–d are provided as a Source Data file.
