# Supplementary material for: Variant-selective stereopure oligonucleotides protect against pathologies associated with C9orf72-repeat expansion in preclinical models
Source: Nat Commun. 2021 Feb 8;12:847. doi: 10.1038/s41467-021-21112-8 (PMC7870851; doi:10.1038/s41467-021-21112-8)
Supplement: Supplementary file 1 — Supplementary Information [file 41467_2021_21112_MOESM1_ESM.pdf]

Supplementary Information

Variant-selective stereopure oligonucleotides protect against pathologies associated with *C9orf72*-repeat expansion in preclinical models

Yuanjing Liu[1,†], Jean-Cosme Dodart[1,†], Helene Tran[2,†], Shaunna Berkovitch[1], Maurine Braun[1], Michael Byrne[1], Ann Durbin[1], Xiao Shelley Hu[1], Naoki Iwamoto[1], Hyun Gyung Jang[1], Pachamuthu Kandasamy[1], Fangjun Liu[1], Kenneth Longo[1], Jörg Ruschel[1], Juili Shelke[1], Hailin Yang[1], Yuan Yin[1], Amy Donner[1], Zhong Zhong[1], Chandra Vargeese[1*], Robert H. Brown, Jr.[2]

Affiliations:

[1]Wave Life Sciences Ltd., Cambridge, MA.

[2]Department of Neurology, University of Massachusetts, Worcester, MA.

*To whom correspondence should be addressed: cvargeese@wavelifesci.com

Supplementary Figures

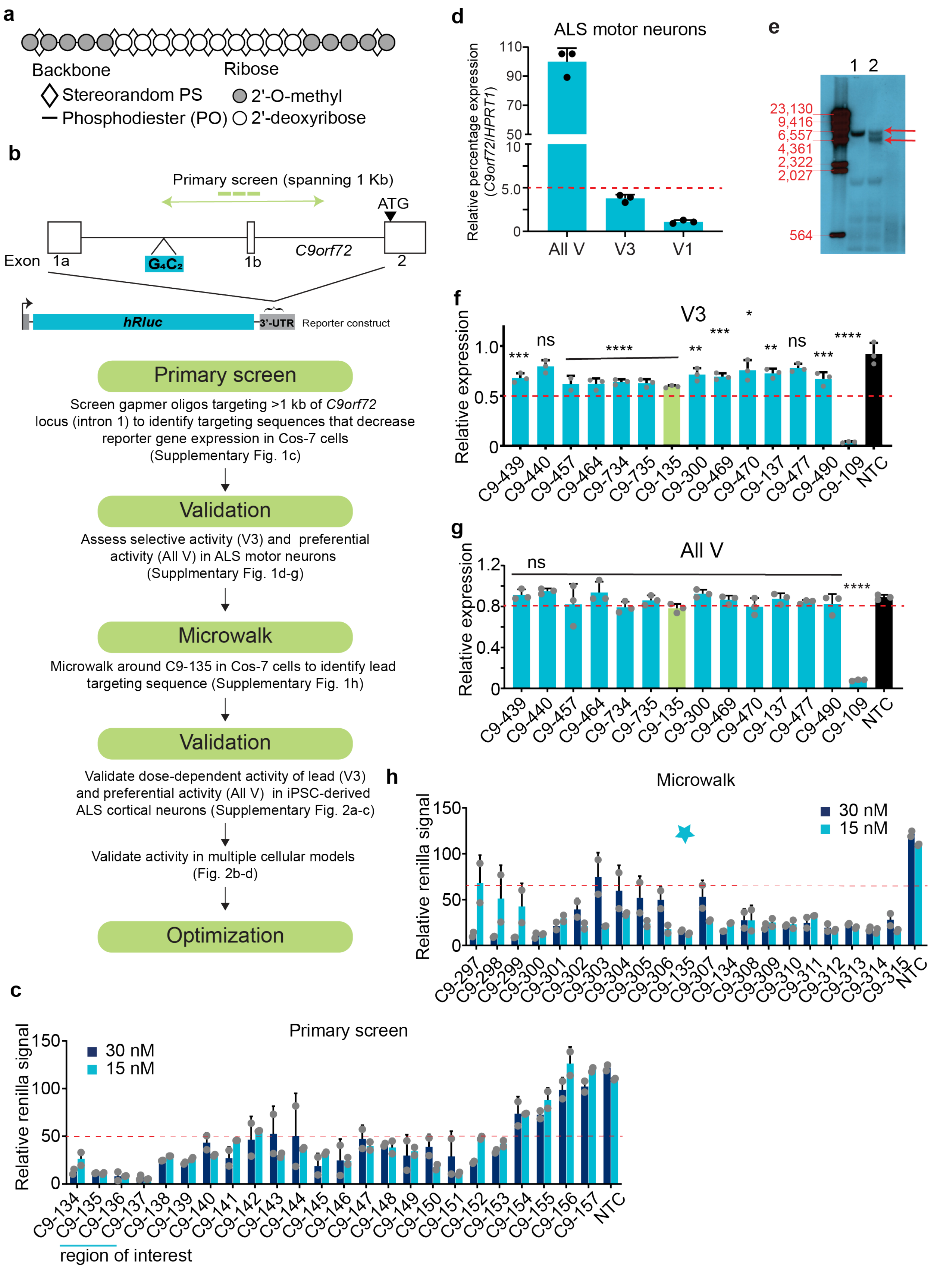
a
Backbone
Ribose
Stereorandom PS
2'-O-methyl
Phosphodiester (PO)
2'-deoxyribose
b
Primary screen (spanning 1 Kb)
ATG
Exon 1a
G4C2
1b
C9orf72
2
hRluc
3'-UTR
Reporter construct
Primary screen
Screen gapmer oligos targeting >1 kb of C9orf72 locus (intron 1) to identify targeting sequences that decrease reporter gene expression in Cos-7 cells (Supplementary Fig. 1c)
Validation
Assess selective activity (V3) and preferential activity (All V) in ALS motor neurons (Supplmentary Fig. 1d-g)
Microwalk
Microwalk around C9-135 in Cos-7 cells to identify lead targeting sequence (Supplementary Fig. 1h)
Validation
Validate dose-dependent activity of lead (V3) and preferential activity (All V) in iPSC-derived ALS cortical neurons (Supplementary Fig. 2a-c)
Validate activity in multiple cellular models (Fig. 2b-d)
Optimization
c
Primary screen
Relative renilla signal
30 nM
15 nM
region of interest
d
ALS motor neurons
Relative percentage expression (C9orf72/HPRT1)
All V
V3
V1
e
1 2
23,130
9,416
6,557
4,361
2,322
2,027
564
f
V3
Relative expression
ns
g
All V
Relative expression
ns
h
Microwalk
Relative renilla signal
30 nM
15 nM

Supplementary Fig. 1. Targeting-site identification. **a** Schematic representation of chemical modifications applied to gapmer oligonucleotides used in the primary screen to identify the targeting sequence. Oligonucleotides have stereorandom backbone chemistry at all phosphorothioate (PS)-modified positions, 2'-O-methyl ribose modifications and three phosphodiester linkages in the wings to confer nuclease stability [1,2]. The 10-nucleotide gap features deoxyribose sugar. **b** Overview of the screening and validation workflow applied to identify SS1b. **c** Data from the primary screen in Cos-7 cells for 23 out of approximately 200 oligonucleotides. Signal from renilla luciferase (standardized to firefly luciferase) is shown for Cos-7 cells treated with 15 or 30 nM of the indicated oligonucleotide. Aqua bar highlights region with most potent activity. Data are presented as mean ± SD, n=2. **d** Relative percentage expression of the indicated *C9orf72* transcript with respect to *HPRT* in human motor neurons derived from ALS iPSCs. V3 and V1 account for ~3.8% and 1.1% of total *C9orf72* transcripts, respectively. Data are presented as mean ± SD, n=3. **e** Southern blot confirming the C9-G_4C_2-repeat expansion is present in ALS iPSCs (lane 1) and ALS motor neurons (lane 2). The red arrows demarcate repeat expansions. This experiment was performed once. Molecular weight markers (base pairs) are indicated. Motor neuron samples reveal two distinct expansions, indicating mixed lineage in the culture. **f** Relative expression of V3 transcripts with respect to *HPRT* for human ALS motor neurons (iPSC-derived) treated under gymnotic conditions with 10 µM of the indicated oligonucleotide. C9orf72-109 targets a sequence in exon 2, and the non-targeting control (NTC, black) is not complementary to *C9orf72.* Data are presented as mean ± SD, n=3. One-way ANOVA with Dunnett's multiple comparisons test: **** P<0.0001, ***P<0.001, ** P<0.01, * P<0.05, ns non-significant. **g** Relative expression of all variants (All V) with respect to *HPRT* in human ALS motor neurons treated and data presented as in f (n=3). **h** Microwalk around C9orf72-135 to validate and refine the targeting sequence. Signal from renilla luciferase (standardized as in c) is shown for Cos-7 cells treated with 15 or 30 nM of the indicated oligonucleotide. The star depicts the location of Splice-site 1b (SS1b). Data are mean ± SD, n=2. Source data, including exact P values for panels f and g, are provided as a Source Data file. Kb, kilobase; NTC, non-targeting control; V, variant

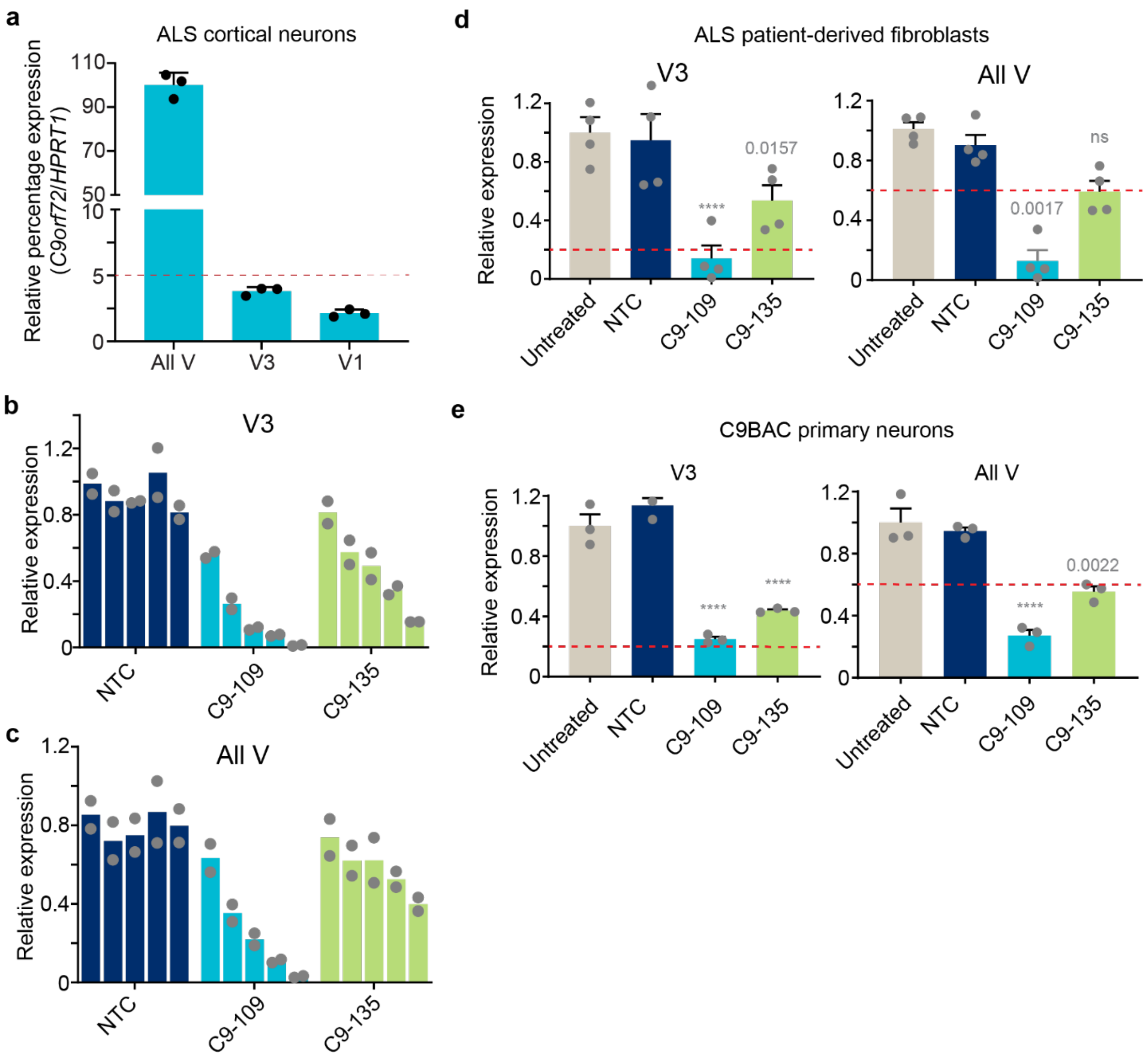

Supplementary Fig. 2. Validation of oligonucleotide activity. **a** Relative percentage expression of the indicated *C9orf72* transcript with respect to *HPRT* in human ALS cortical neurons. V3 and V1 account for ~3.8% and 2.1% of total *C9orf72* transcripts, respectively. Data are presented as mean ± SD, n=3. **b** Relative expression of V3 transcripts with respect to *HPRT* for human ALS cortical neurons treated under gymnotic conditions for two weeks with increasing concentrations (100 nM-10 µM) of the indicated oligonucleotide. Data are presented as means, n=2. **c** Relative expression of all variants (All V) with respect to *HPRT* in human ALS cortical neurons under the same conditions as in b. Data are presented as means, n=2. **d** Relative expression of the indicated *C9orf72* transcript (V3, left; all variants, right) with respect to *HPRT* for ALS patient-derived fibroblasts transfected with 100 nM of the indicated oligonucleotide. Data are presented as mean ± SEM, n=4. One-way ANOVA with Dunnett's multiple comparison's test ****, P<0.0001, ns, non-significant, or exact P value is indicated. **e** Relative expression of the indicated *C9orf72* transcript (V3, left; all variants, right) with respect to *Hprt* for mouse C9BAC primary neurons treated under gymnotic conditions with 1 µM of the indicated oligonucleotide. Data are presented as mean ± SEM, n=3. One-way ANOVA with Dunnett's multiple comparisons, **** P<0.0001 or exact P value is indicated. Source data are provided as a Source Data file. All V, All variants; NTC, non-targeting control.

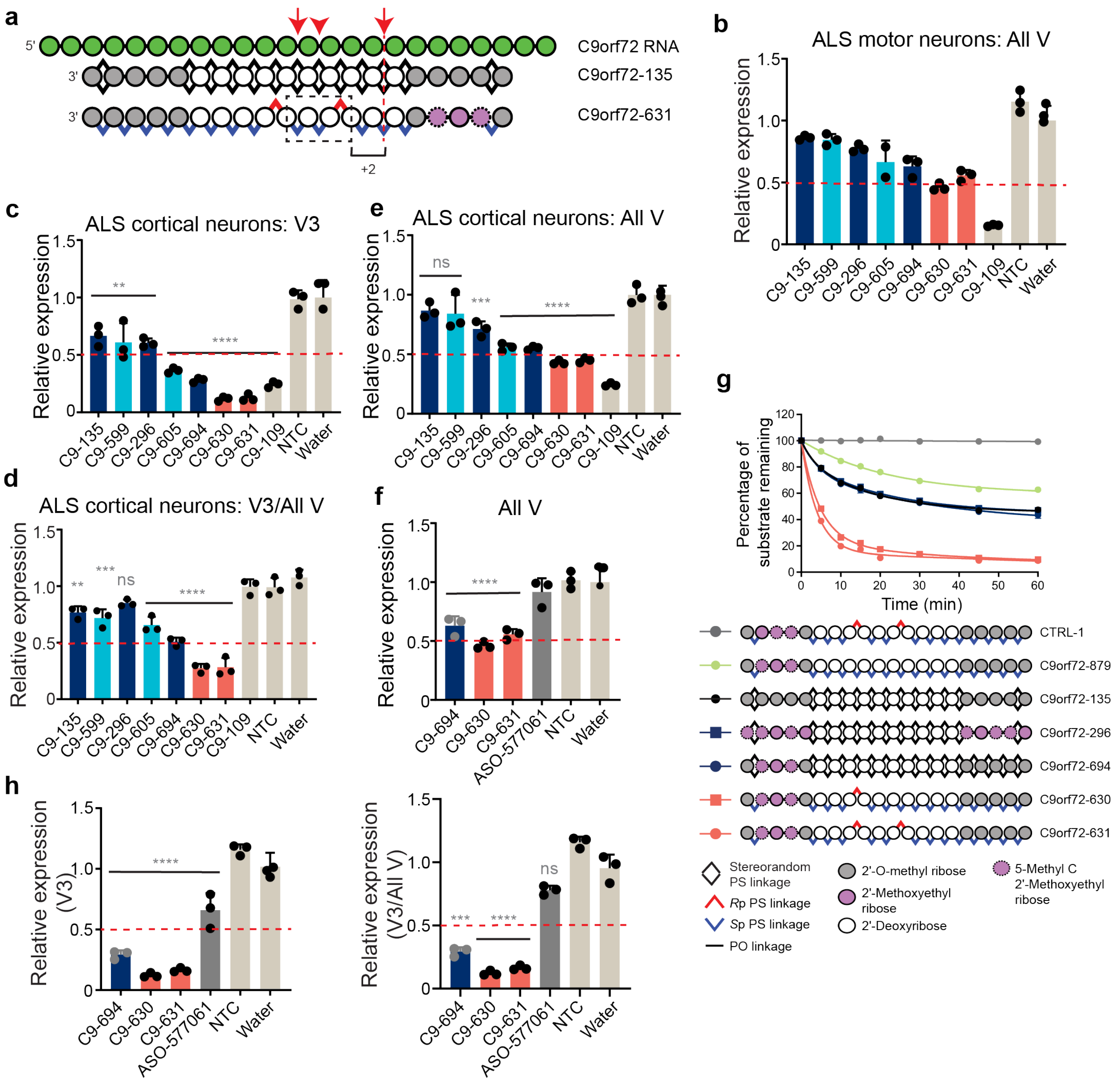

Supplementary Fig 3. C9orf72-630 and C9orf72-631 outperform other oligonucleotides in biochemical and cellular assays. **a** Strategy for positioning the SSR motif. In the cartoon, C9orf72 RNA is a surrogate RNA. C9-135 and C9-631 are shown. Red arrows indicate major and minor cleavage sites in RNA. The dotted black box highlights first SSR motif. The dotted red line and +2 help illustrate the relationship between the SSR motif and the cleavage position. In RNase H1 biochemical experiments, the stereorandom oligonucleotide (C9-135) yields two major and one minor cleavage site in surrogate C9orf72 RNA. Placement of the 3'-SSR-5' motifs is dictated by this cleavage pattern. The 5'-most SSR motif is positioned to direct cleavage at +2 position in alignment with pre-existing cleavage preferences[3]. The additional SSR motif is positioned to promote cleavage within the other RNase H-sensitive region. Changes in wing chemistry from symmetric 2'-OMe (C9-135) to symmetric 2'-MOE (C9-296) or asymmetric (C9-694) yield the same predominant cleavage positions, so they do not impact positioning of the SSR motif. **b** Relative expression of all variants with respect to *HPRT* in human ALS motor neurons after gymnotic treatment with 10 µM of the indicated ASOs (stereorandom, navy; stereopure, aqua; stereopure with asymmetric wing chemistry, coral; negative controls, beige). Data are presented as mean ± SD, n=3. Dotted horizontal line denotes 0.5-fold expression level in all bar graphs. **c** Relative expression of V3 with respect to *HPRT* in human ALS cortical neurons after one week of gymnotic treatment with 10 µM of the indicated ASOs. Data are presented as in

b (n=3). One-way ANOVA with Dunnett's multiple comparisons ** P<0.01 (C9-135, P=0.0069; C9-599, P=0.0012; C9-296, P=0.0011), **** P<0.0001. **d** Ratio of V3:All V with respect to *HPRT* in human ALS cortical neurons after gymnotic treatment with 10 µM of the indicated ASOs (stereorandom, navy; stereopure, aqua; stereopure with asymmetric wing chemistry, coral; negative controls, beige). Data are presented as mean ± SD, n=3. One-way ANOVA with Dunnett's multiple comparisons **, P=0.0027; ***, P=0.0003; **** P<0.0001; ns, non-significant. **e** Relative expression of all variants with respect to *HPRT* in human ALS cortical neurons, and data are presented as in b. One-way ANOVA with Dunnett's multiple comparisons ns, non-significant, *** P=0.003, **** P<0.0001. **f** Relative expression of all variants with respect to *HPRT* in human ALS motor neurons treated with 10 µM of the indicated oligonucleotide. Data are presented as mean ± SD, n=3. One-way ANOVA with Tukey's multiple comparisons. **** P<0.0001. **g** Percentage of *C9orf72* surrogate RNA substrate remaining in biochemical RNase H1 assays over time are plotted for the indicated oligonucleotides. Data are presented as mean ± SEM, and in most cases, error bars are smaller than the symbol, n=3. **h** Relative expression of V3 (left) and the ratio of V3 to all variants (right) with respect to *HPRT* in human ALS motor neurons treated with 10 µM of the indicated oligonucleotide. Data are presented as mean ± SD, n=3. One-way ANOVA with Tukey's multiple comparisons. **** P<0.0001, *** P=0.0003 (C9-694). Source data, including exact P values for panels d, are provided as a Source Data file. NTC, non-targeting control; All V, All Variants; PS phosphorothioate; PO, phosphodiester; C, cytosine

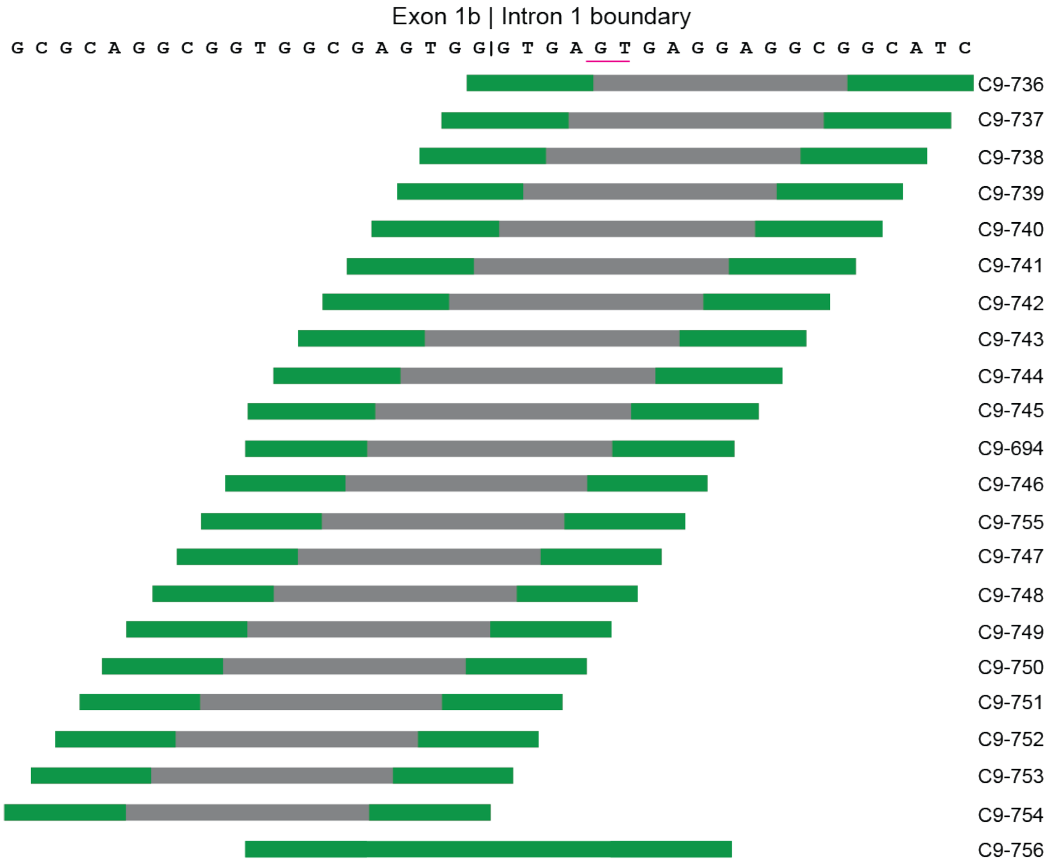

Supplementary Figure 4. Sequence of the boundary region between exon 1b and intron 1 in the *C9orf72* gene (sequence shown is DNA) that contains SS1b. Oligonucleotides used in the microwalk shown in Fig. 3b are aligned to show their respective complementarities to this region. Oligonucleotide names are indicated to the right, and they are illustrated 3'-5'. Green regions represent wings containing 2'-sugar modified nucleotides. Gray regions represent the DNA gaps. In the sequence, '|' represents the boundary between exon and intron, and the sequences underlined in pink indicate the 5'-splice site.

a

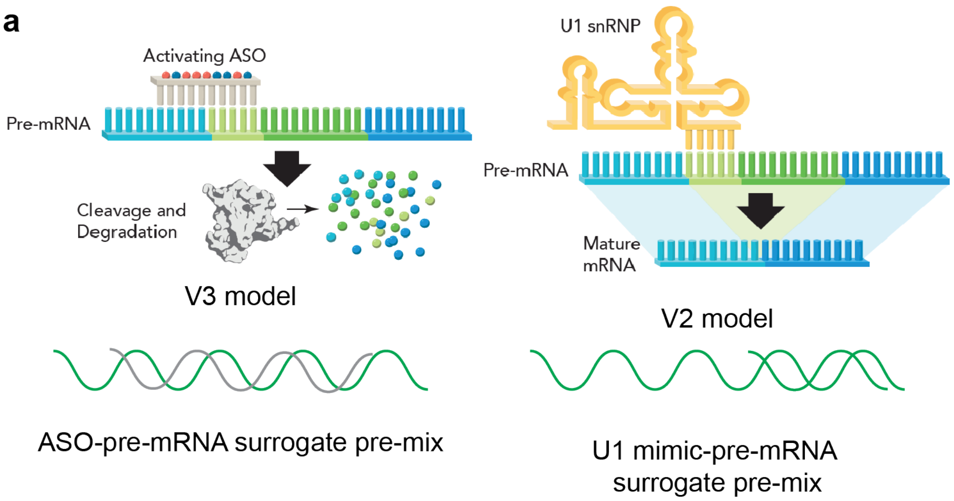

b

Complex	Tm (°C)	Peak molarity (nmol/L)
C9-631/RNA surrogate	77.7	2,250
U1 mimic/RNA surrogate	25.8	Not detected
C9-631-U1/RNA surrogate	77.6	3,290

c

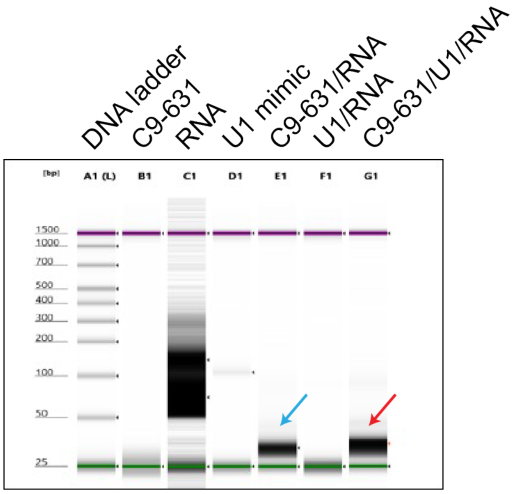

Supplementary Fig. 5. Biochemical assessment of U1 mimic and C9orf72-631 competition for a *C9orf72* RNA surrogate. **a** Model illustrating rationale for experiments. By pre-mixing oligonucleotide (gray) with surrogate pre-mRNA (green), we mimic the expected conditions for V3 transcripts in cells (left panel). By pre-mixing the U1 mimic with surrogate pre-mRNA (green), we mimic the expected conditions for V2 transcripts in cells, where the spliceosome is expected to bind (right panel). **b** Measured Tms and peak molarity for the complexes: heteroduplex C9orf72-631/RNA, heteroduplex U1 mimic/RNA and mixed heteroduplexes or triplex C9orf72-631/U1/RNA. **c** Electrophoretic evaluation of complex formation. C9orf72-631/RNA heteroduplex is smaller than the C9orf72-631/U1/RNA complex. Peak molarity ratio of heteroduplex: heterotriplex is 1.5. Molecular weight markers (base pairs) are shown. This experiment was performed once.

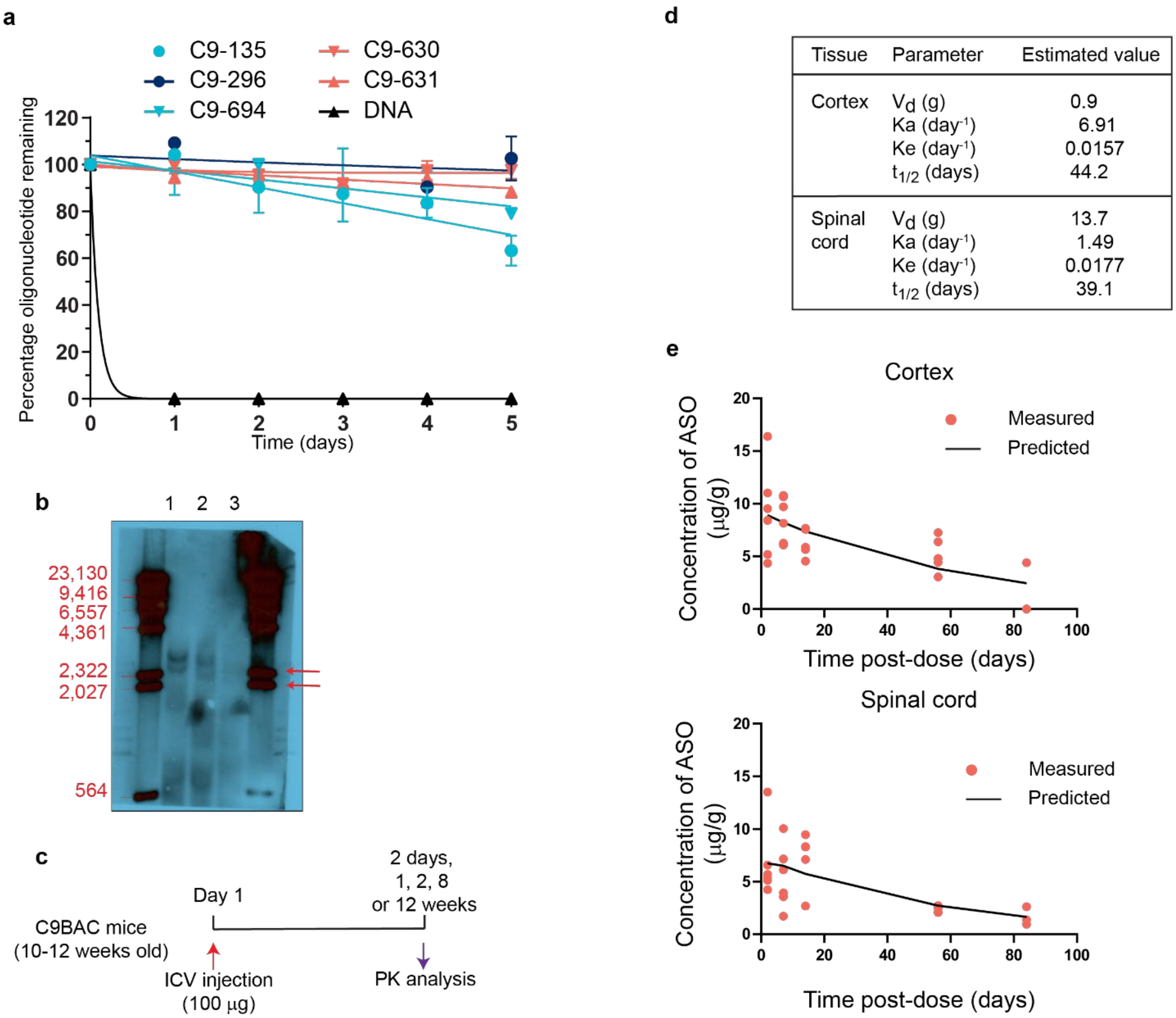

Tissue	Parameter	Estimated value
Cortex	V_d (g)	0.9
	Ka (day^{-1})	6.91
	Ke (day^{-1})	0.0157
	$t_{1/2}$ (days)	44.2
Spinal cord	V_d (g)	13.7
	Ka (day^{-1})	1.49
	Ke (day^{-1})	0.0177
	$t_{1/2}$ (days)	39.1

Supplementary Fig. 6. *Ex vivo* oligonucleotide stability and pharmacokinetic (PK) study in C9BAC mice. **a** The percentage of full-length oligonucleotide remaining after incubation in mouse brain homogenate *ex vivo* is plotted with respect to incubation time (days) for the indicated oligonucleotides and for DNA control oligonucleotide (5'-GGCACAAGGGCACAGACTTC). Data are presented as mean ± SD, n=3. Lines represent 1-phase decay least-squares fit of data. **b** Southern blot confirming C9-G_4C_2-repeat expansion is present in C9BAC transgenic mice. Molecular weight markers (base pairs) are indicated in red. Genomic DNA from three different mice are shown (lanes, 1, 2 and 3). Repeat expansions are indicated by the red arrows. The upper band contains 500 and the lower band contains 300 repeats. This experiment was performed once. **c** Schematic representation of dosing regimen for 12-week PK study. **d** PK parameters estimated in C9BAC mice. **e** Concentration of oligonucleotide in cortex (top) and spinal cord (bottom) are plotted with respect to time post-dose (days). Measured and predicted values are shown. Source data are provided as a Source Data file. ASO, antisense oligonucleotide; Vd, apparent volume of distribution; Ka, absorption rate constant; Ke, elimination rate constant; $t_{1/2}$, half-life.

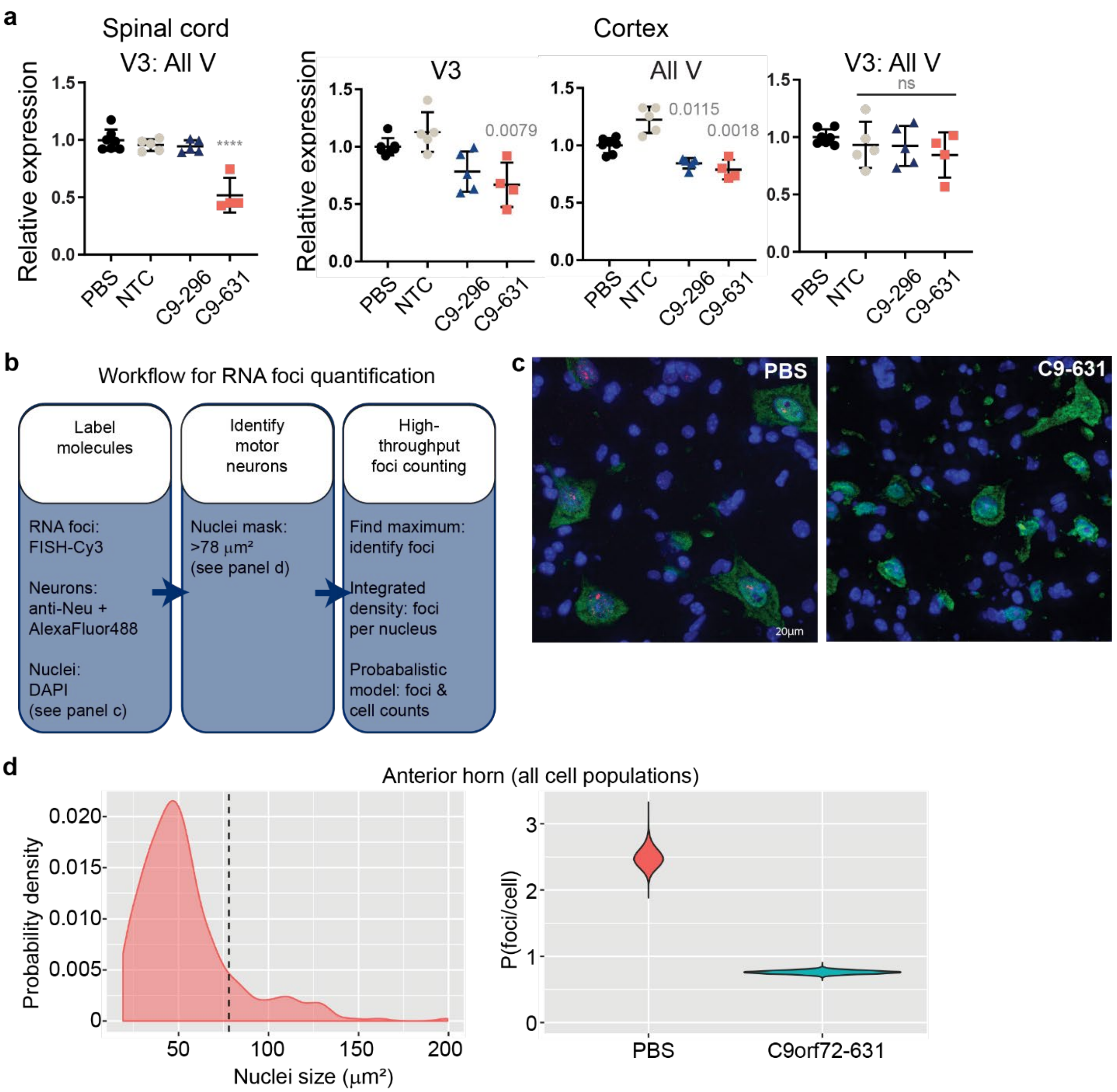

Supplementary Fig. 7. Target engagement and resolution of pathological features in C9BAC mouse 2-week study. **a** Relative expression levels for the indicated transcripts in the spinal cord (left) and cortex (right) are shown for the indicated oligonucleotide. Transcript expression is standardized to *Hprt*, individual points indicate data from one mouse, and data are presented as mean ± SD (PBS, n=7; NTC and C9-296, n=5; C9-631, n=4). One-way ANOVA with Dunnett's multiple comparisons. **** P<0.0001; ns, non-significant, or exact P value is indicted on graph. **b** Overview of the workflow for quantification of RNA foci. **c** Representative, wide-field, merged images from evaluation of 4 mice per treatment group showing spinal cords of mice treated with PBS (left) or C9orf72-631 (right) and stained for RNA foci (Cy3-labeled probes, red), nuclei (DAPI, blue) and neurons (anti-NeuN, green). 20 µm scale bars are shown. **d** Violin plot showing size distribution of nuclei detected in anterior horn images, indicating the presence of multiple cell types with distinct nuclei sizes. Dotted line (70 µm^2) demarcates cut off for motor neuron nuclei, which are large compared with other cell types. Motor neurons were defined as cells present in the anterior horn of the spinal cord that are both NeuN-positive have nuclei that are larger than the indicated cutoff. Right panel shows quantification of RNA foci per cell (including all cells) of the anterior horn in mice treated

with PBS (fuschia) or C9-631 (aqua). Source data, including exact P values for panel a, are provided as a Source Data file. P, probability; PBS, phosphate-buffered saline; V, variant; FISH, fluorescence *in situ* hybridization.

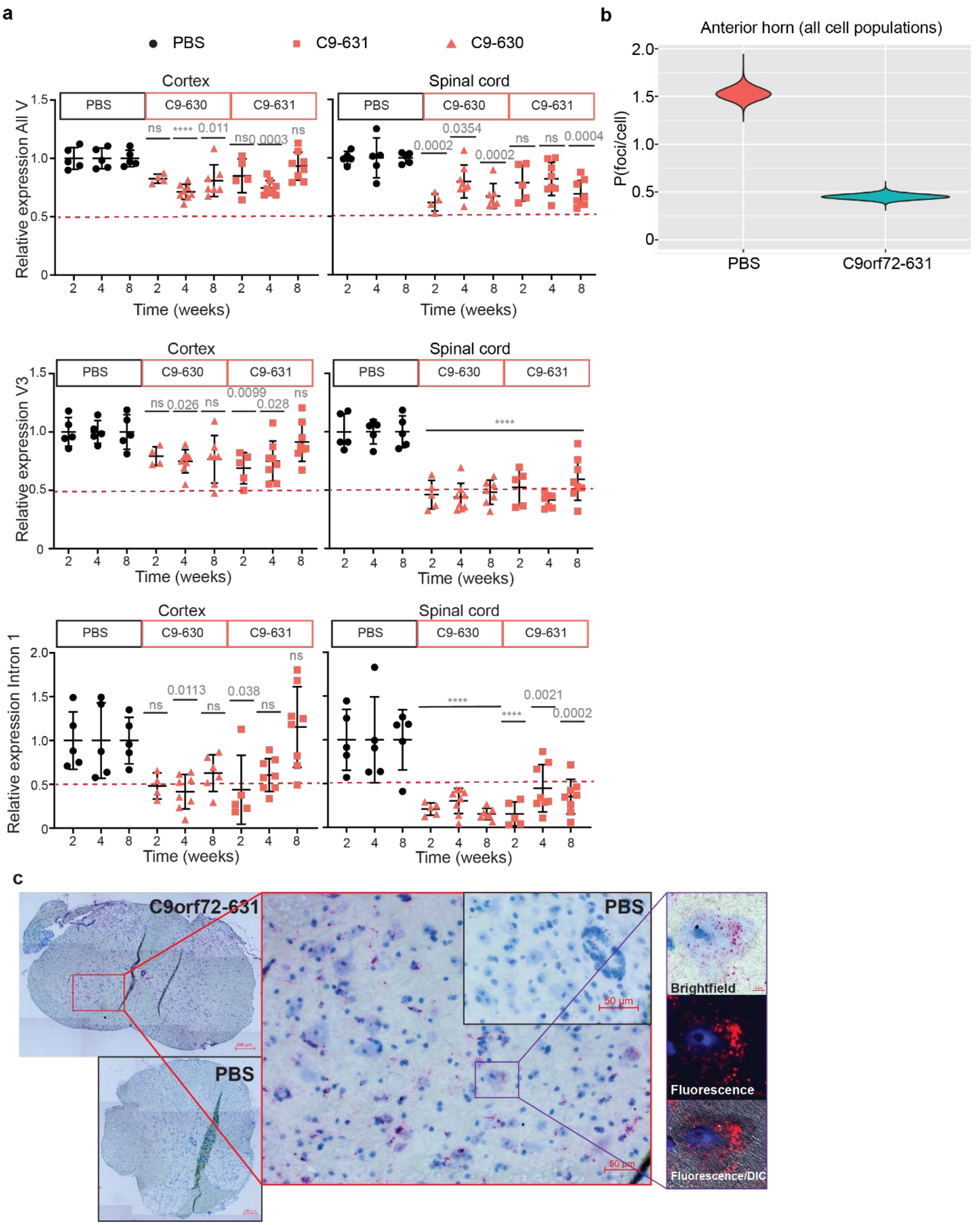

Supplementary Fig. 8. C9orf72-631 penetrates motor neurons in the CNS and decreases the prevalence of RNA foci for up to 8 weeks. a Relative transcript levels of all variants (top), V3 (middle) or intron 1-containing transcripts (bottom) in cortex (left) or spinal cord (right) at 2-, 4-, and 8- weeks post-dose are shown for PBS- (black), C9orf72-630- (coral triangles), C9orf72-631- (coral squares) treated mice. Data are presented as mean ± SD

[PBS, n=5 all time points; C9-630, n=5 (2 weeks) n=7 (4 and 6 weeks), and C9-631, n=8 all time points]. Two-way ANOVA with Student-Newman-Keuls multiple comparisons, ****, $P<0.0001$ or P values indicated on graph. **b** Quantification of RNA foci per cell (including all cells) of the anterior horn in mice treated with PBS (fuschia) or C9orf72-631 (aqua). **c** View RNA detection of oligonucleotide in spinal cord motor neurons in C9orf72-631-treated but not PBS-treated C9BAC mice. Representative bright field images from evaluation of 8 animals treated with C9-631 and 6 animals treated with PBS across 3 experiments are shown at 10X magnification (left, 100 μm scale bar), with 20X magnification (middle, 50 μm scale bar) of C9orf72-631 sample or PBS sample (inset), and >63X magnification (right, 5 μm scale bar) in bright field (top), fluorescence field (middle) and overlay of fluoresence/differential interference contrast (DIC). Nuclei are blue (hematoxylin and Hoechst 33342, respectively) and oligonucleotide is red (View RNA and Cy3, respectively). Source data are provided as a Source Data file. PBS, phosphate-buffered saline

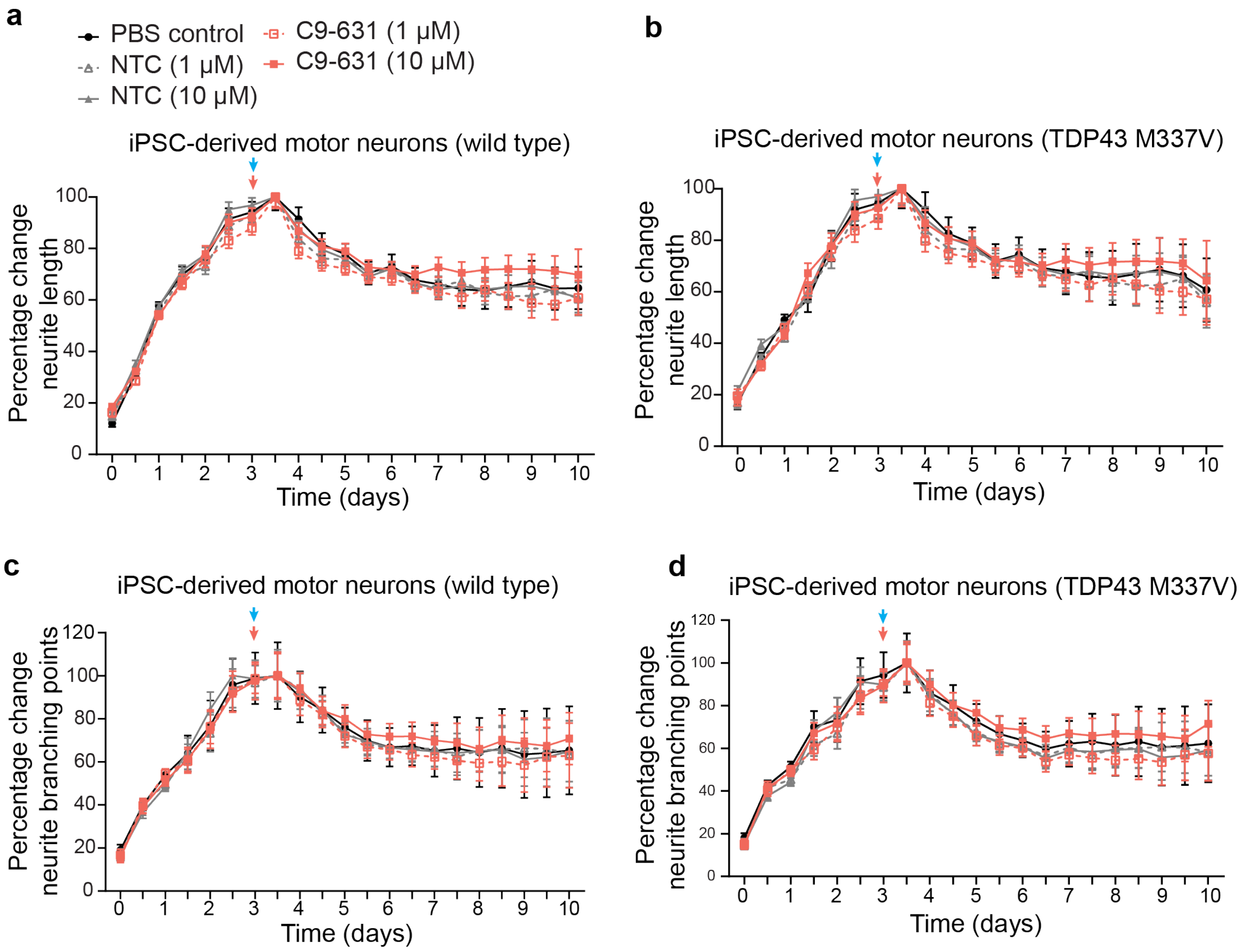

Supplementary Fig. 9. Human iPSC-derived motor neurons lacking C9orf72-repeat expansion are not rescued by C9orf72-631. Percentage change in neurite length (**a,b**) and neurite branching points (**c,d**) over time (days) for wild-type (**a,c**) or *TARDBP* M337V heterozygous (**b,d**) motor neurons treated as shown in Fig. 6a. For a-d, data are presented as mean ± SEM, n=3 per time point. Source data, including exact P values, are provided as a Source Data file. Treated samples (C9orf72-631) and control (NTC)-treated samples were not significantly different from untreated, Two-way ANOVA with Dunnett's multiple comparisons.

A

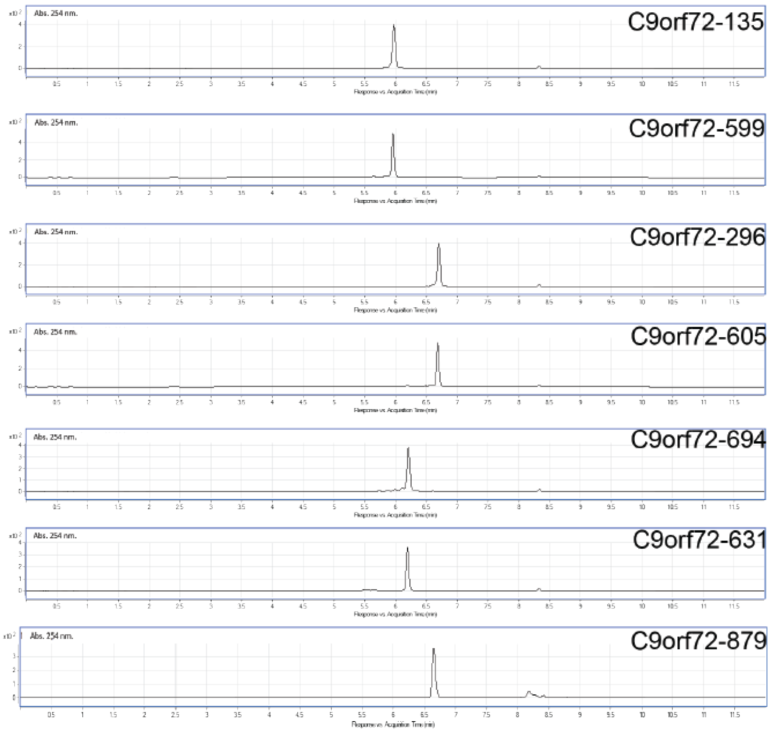

B

Name	Mass	
	Observed	*Calculated*
C9orf72-135	6397.755	6397.915
C9orf72-599	6397.765	6397.915
C9orf72-296	6936.125	6336.627
C9orf72-605	6936.124	6336.627
C9orf72-694	6619.829	6620.349
C9orf72-631	6619.817	6620.349
C9orf72-879	6619.834	6620.349

Supplementary Fig. 10. Characterization of stereopure oligonucleotides. a HPLC chromatograms for stereopure oligonucleotides. **b** Observed and calculated masses for stereopure oligonucleotides.

Supplementary Tables

Supplementary Table 1. The sequence and chemistry of oligonucleotides used in this study.

Name	Sequence & Chemistry
C9orf72-134	UCACUCACCCACTCGCCACC
C9orf72-135	CCUCACTCACCCACTCGCCA
C9orf72-136	GGAUGCCGCCTCCTCACUCA
C9orf72-137	GCCAGGATGCCGCCTCCUCA
C9orf72-138	CCCCAAACAGCCACCCGCCA
C9orf72-139	GAGAGCCCCCGCTTCUACCC
C9orf72-140	GCUCUGAGGAGAGCCCCCGC
C9orf72-141	GAGCUCTGAGGAGAGCCCCC
C9orf72-142	GAUCCCCATCCCTTGUCCCU
C9orf72-143	GCCAGATCCCCATCCCUUGU
C9orf72-144	GAGGCCAGATCCCCAUCCCU
C9orf72-145	GGCUCCCTTTTCTCGAGCCC
C9orf72-146	GUACCCGAGGCTCCCUUUUC
C9orf72-147	CUCAGTACCCGAGGCUCCCU
C9orf72-148	UCUCAGTACCCGAGGCUCCC
C9orf72-149	GCCUCTCAGTACCCGAGGCU
C9orf72-150	GGCCUCTCAGTACCCGAGGC
C9orf72-151	CCUCCGGCCTTCCCCCAGGC
C9orf72-152	ACCCUCCGGCCTTCCCCCAG
C9orf72-153	CCACCCTCCGGCCTTCCCCC
C9orf72-154	ACCCCCATCTCATCCCGCAU
C9orf72-155	CACCCCCATCTCATCCCGCA
C9orf72-156	GGCGUCTCCACACCCCCAUC
C9orf72-157	GCAGGCGTCTCCACACCCCC

Stereorandom phosphorothioate (PS) backbone ◇, Phosphodiester (PO) backbone —,
2'-O-methyl ribose(2'-OMe) ●, 2'-deoxyribose (DNA) ○

Table 1, continued

Name	Sequence & Chemistry
C9orf72-297	GAUGCCGCCTCCTCACUCAC
C9orf72-298	AUGCCGCCTCCTCACUCACC
C9orf72-299	UGCCGCCTCCTCACTCACCC
C9orf72-300	GCCGCCTCCTCACTCACCCA
C9orf72-301	CCGCCTCCTCACTCACCCAC
C9orf72-302	CGCCUCCTCACTCACCCACU
C9orf72-303	GCCUCCTCACTCACCCACUC
C9orf72-304	CCUCCTCACTCACCCACUCG
C9orf72-305	CUCCUCACTCACCCACUCGC
C9orf72-306	UCCUCACTCACCCACUCGCC
C9orf72-307	CUCACTCACCCACTCGCCAC
C9orf72-308	CACUCACCCACTCGCCACCG
C9orf72-309	ACUCACCCACTCGCCACCGC
C9orf72-310	CUCACCCACTCGCCACCGCC
C9orf72-311	UCACCCACTCGCCACCGCCU
C9orf72-312	CACCCACTCGCCACCGCCUG
C9orf72-313	ACCCACTCGCCACCGCCUGC
C9orf72-314	CCCACTCGCCACCGCCUGCG
C9orf72-315	CCACUCGCCACCGCCUGCGC

Stereorandom PS backbone ◊, PO backbone —,
2'-OMe ribose ●, DNA ○

Table 1, continued

Name	Sequence & Chemistry
C9orf72-439	GUUCACCCTCAGCGAGUACU
C9orf72-440	CUUGUTCACCCTCAGCGAGU
C9orf72-457	GAGCUTGCTACAGGCUGCGG
C9orf72-464	GCGCGACTCCTGAGTUCCAG
C9orf72-734	CCCGGCCCCTAGCGCGCGAC
C9orf72-735	UACAGGCTGCGGTTGUUUCC
C9orf72-469	CAGGATGCCGCCTCCUCACU
C9orf72-470	CCAGGATGCCGCCTCCUCAC
C9orf72-477	GCCACCCGCCAGGATGCCGC
C9orf72-490	CGCCUCTTCCCGGCAGCCGA
C9orf72-109	GUGCUGCGATCCCCAUUCCA
C9orf72-599	CCUCACTCACCCACTCGCCA
C9orf72-296	CCTCACTCACCCACTCGCCA
C9orf72-605	CCTCACTCACCCACTCGCCA
C9orf72-694	CCTCACTCACCCACTCGCCA
C9orf72-631	CCTCACTCACCCACTCGCCA

Stereorandom PS backbone ◊ , PO backbone — ,
2'-OMe ribose ● , DNA ○, 2'-Methoxyethyl ribose (2'-MOE) ● , 5-Methyl C, 2'-MOE
Rp PS backbone ^, *Sp* PS backbone V

Table 1, continued

Name	Sequence & Chemistry
C9orf72-736	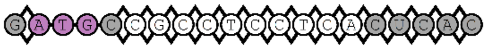
C9orf72-737	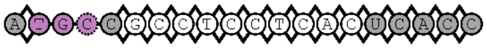
C9orf72-738	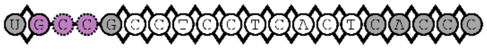
C9orf72-739	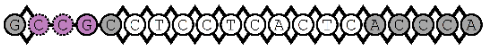
C9orf72-740	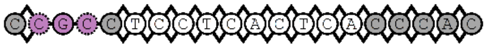
C9orf72-741	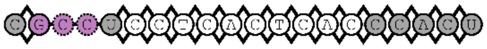
C9orf72-742	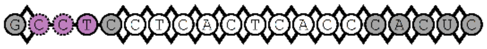
C9orf72-743	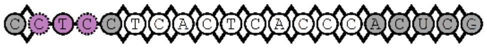
C9orf72-744	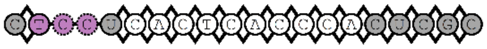
C9orf72-745	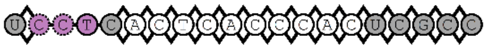
C9orf72-746	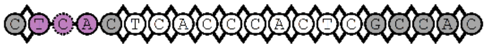
C9orf72-755	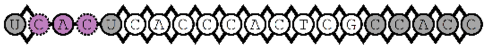
C9orf72-747	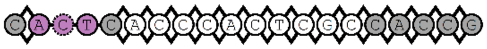
C9orf72-748	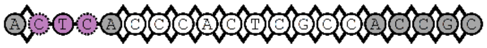
C9orf72-749	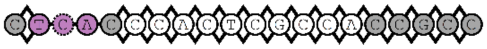
C9orf72-750	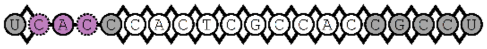
C9orf72-751	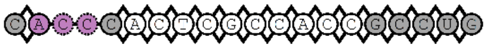
C9orf72-752	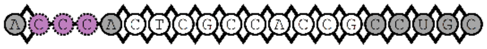
C9orf72-753	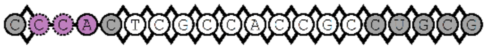
C9orf72-754	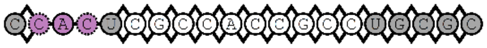
C9orf72-756	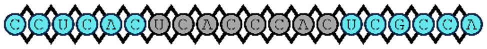

Stereorandom PS backbone ◊, PO backbone —,
2'-OMe ribose ●, DNA ○, 2'-MOE ●, 5-Methyl C, 2'-MOE ●,
Rp PS backbone ^, *Sp* PS backbone V, 2'-fluoro ribose ●

Table 1, continued

Name	Sequence & Chemistry
ASO-577061	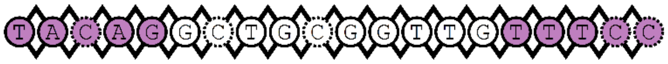
ASO1	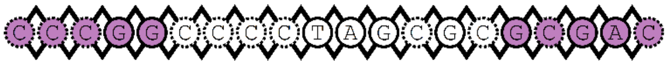
ASO5	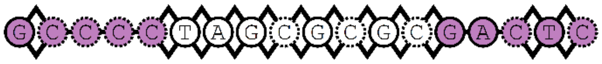

Stereorandom PS backbone ◊, PO backbone —,
DNA ○, 2’-Methoxyethyl ●, 5-Methyl C 2’-Methoxyethyl ●

Primer name	Primer sequence
**Intron1 forward primer**	GGTCAGAGAAATGAGAGGGAAAG
**Intron1 reverse primer**	CGAGTGGGTGAGTGAGGA
**Intron 1 probe (FAM)**	AAATGCGTCGAGCTCTGAGGAGAG
**Mouse Hprt forward primer**	CAAACTTTGCTTTCCCTGGTT
**Mouse Hprt reverse primer**	TGGCCTGTATCCAACACTTC
**Mouse Hprt probe (HEX)**	ACCAGCAAGCTTGCAACCTTAACC

Supplementary Table 2. List of primers used in this work.

Supplementary References

1 Tabrizi, S. J., Ghosh, R. & Leavitt, B. R. Huntingtin Lowering Strategies for Disease Modification in Huntington's Disease. *Neuron* **102**, 899, doi:10.1016/j.neuron.2019.05.001 (2019).

2 Graham, M. J. *et al.* Cardiovascular and Metabolic Effects of ANGPTL3 Antisense Oligonucleotides. *The New England journal of medicine* **377**, 222-232, doi:10.1056/NEJMoa1701329 (2017).

3 Iwamoto, N. *et al.* Control of phosphorothioate stereochemistry substantially increases the efficacy of antisense oligonucleotides. *Nature biotechnology* **35**, 845-851, doi:10.1038/nbt.3948 (2017).
