# Supplementary material for: Variant-selective stereopure oligonucleotides protect against pathologies associated with C9orf72-repeat expansion in preclinical models
Source: Nat Commun. 2021 Feb 8;12:847. doi: 10.1038/s41467-021-21112-8 (PMC7870851; doi:10.1038/s41467-021-21112-8)
Supplement: Supplementary file 2 — Reporting Summary [file 41467_2021_21112_MOESM2_ESM.pdf]

nature research

Corresponding author(s): Chandra Vargeese

Last updated by author(s): 12/17/2020

Reporting Summary

Nature Research wishes to improve the reproducibility of the work that we publish. This form provides structure for consistency and transparency in reporting. For further information on Nature Research policies, see Authors & Referees and the Editorial Policy Checklist .

Statistics

For all statistical analyses, confirm that the following items are present in the figure legend, table legend, main text, or Methods section.

n/a	Confirmed	
☐	☒	The exact sample size (*n*) for each experimental group/condition, given as a discrete number and unit of measurement
☐	☒	A statement on whether measurements were taken from distinct samples or whether the same sample was measured repeatedly
☐	☒	The statistical test(s) used AND whether they are one- or two-sided<br>*Only common tests should be described solely by name; describe more complex techniques in the Methods section.*
☐	☒	A description of all covariates tested
☐	☒	A description of any assumptions or corrections, such as tests of normality and adjustment for multiple comparisons
☐	☒	A full description of the statistical parameters including central tendency (e.g. means) or other basic estimates (e.g. regression coefficient) AND variation (e.g. standard deviation) or associated estimates of uncertainty (e.g. confidence intervals)
☐	☒	For null hypothesis testing, the test statistic (e.g. *F*, *t*, *r*) with confidence intervals, effect sizes, degrees of freedom and *P* value noted<br>*Give P values as exact values whenever suitable.*
☐	☒	For Bayesian analysis, information on the choice of priors and Markov chain Monte Carlo settings
☒	☐	For hierarchical and complex designs, identification of the appropriate level for tests and full reporting of outcomes
☒	☐	Estimates of effect sizes (e.g. Cohen's *d*, Pearson's *r*), indicating how they were calculated

Our web collection on statistics for biologists contains articles on many of the points above.

Software and code

Policy information about availability of computer code

Data collection	LI-COR Biosciences Image studio V5.2 to quantify western blots,<br>MESO QUICKPLEX SQ 120 Reader Discovery workbench 4.0.12 (LSR_4_0_12) to quantify poly GP in tissue samples,<br>4200 Agilent TapeStation for collection of biochemical RNase H duplex information software A.02.02,<br>Acquisition of luciferase data: Molecular Devices Spectramax M5 with Softmax pro 7.0 software<br>Acquisition and analysis of oligonucleotide stability data using Xcalibur TM (version 4.0.27.10, ThermoFisher Scientific),<br>Bio-Rad CFX manager 3.1 for qPCR with BioRad CFX Maestro 1.1 version 4.1.2433.1219 software<br>Incucyte S3 2017A<br>SYNERGY HTX plate reader with Gen 5 3.09 software for neurite assays<br>Zen 2.3 software for View RNA data collection<br>Spinning Disk microscope image capture software MetaMorph version 7.10.3.279 (Molecular Devices) for RNA foci detection
Data analysis	GraphPad Prism 8 software for graphing and statistical analyses (cellular data),<br>R::Stats for RNA foci quantification,<br>Pharmacokinetic parameters assessed using Phoenix® WinNonlin® 8.1 software program (Certara),<br>Sigma Plot 13.0 for ANOVA (in vivo data analyses),<br>Analysis of 4200 Agilent TapStation data with High Sensitivity D1000 screentape<br>IncuCyte S3 2017A<br>Image J 1.50i for RNA foci analysis

For manuscripts utilizing custom algorithms or software that are central to the research but not yet described in published literature, software must be made available to editors/reviewers. We strongly encourage code deposition in a community repository (e.g. GitHub). See the Nature Research guidelines for submitting code & software for further information.

Data

Policy information about availability of data

All manuscripts must include a data availability statement. This statement should provide the following information, where applicable:

- Accession codes, unique identifiers, or web links for publicly available datasets
- A list of figures that have associated raw data
- A description of any restrictions on data availability

The source data underlying Figs 2b-d, 3b-d, 4b-c, 4e-f, 5b, 5d-f, 6c-d, and 6f, and Supplementary Figs 1c-d, 1f-h, 2a-e, 3b-h, 6a, 6e, 7a, 7d, 8a, 8b, 9a-d are provided as a Source Data file.

Field-specific reporting

Please select the one below that is the best fit for your research. If you are not sure, read the appropriate sections before making your selection.

[x] Life sciences [ ] Behavioural & social sciences [ ] Ecological, evolutionary & environmental sciences

For a reference copy of the document with all sections, see nature.com/documents/nr-reporting-summary-flat.pdf

Life sciences study design

All studies must disclose on these points even when the disclosure is negative.

Sample size	Most in vitro experiments performed in this work are standard in our laboratories, and the sample sizes sufficient to yield statistically significant outcomes were established before these experiments were performed. For mouse experiments, preliminary analyses provided insight into the variability of assays, and we designed experiments based on these preliminary observations. For neurite growth studies, preliminary experiments were performed to assess assay variability prior to designing the final experiments. No statistics were used to determine sample sizes. Sample sizes were sufficient because outcomes were readily replicated across new experiments using the same or overlapping oligonucleotides.
Data exclusions	For mouse samples, if the level of housekeeping transcripts (the control RNA) was too low (indicating sample degradation), samples from that mouse were not used. A dye-based evaluation was performed to ensure appropriate cannula placement for ICV injections. If the cannula was misplaced, data from that animal were not used. These exclusion criteria are standard in our laboratories and were established before the onset of this work.
Replication	We performed 5 in vivo studies evaluating overlapping sets of oligonucleotides in overlapping regimens (timing and dose of adminstration and data collection). Data from these studies were generally consistent. In vitro assays to develop C9-630 and C9-631 were performed in 5 C9orf72-expansion-containing cell lines, with assays performed in ALS motor neurons, patient-derived fibroblasts and C9BAC primary neurons performed at least twice. Experiments to evaluate protection of motor neurons against glutamate-induced toxicity were performed twice.
Randomization	Mice were randomized by sex and age into treatment groups.
Blinding	For initial screens, samples were run and processed with the user having no knowledge of which oligonucleotides would yield activity. For in vitro assays, the experimenter was not blinded. It would take multiple investigators - at least one to set up the experiment and one to perform it to achieve blinding. In many assays, for example time sensitive evaluations of RNase H activity or oligonucleotide stability, this is not practical. For others, this would require staffing resources that we do not have. The scientists who performed ICV injections and processed samples from animal experiments were blinded to treatment.

Reporting for specific materials, systems and methods

We require information from authors about some types of materials, experimental systems and methods used in many studies. Here, indicate whether each material, system or method listed is relevant to your study. If you are not sure if a list item applies to your research, read the appropriate section before selecting a response.

Materials & experimental systems

n/a	Involved in the study	
[ ]	[x]	Antibodies
[ ]	[x]	Eukaryotic cell lines
[x]	[ ]	Palaeontology
[ ]	[x]	Animals and other organisms
[ ]	[x]	Human research participants
[x]	[ ]	Clinical data

Methods

n/a	Involved in the study	
[x]	[ ]	ChIP-seq
[x]	[ ]	Flow cytometry
[x]	[ ]	MRI-based neuroimaging

Antibodies

Antibodies used	anti-digoxigenin antibody (Catalog No. 11093274910, Roche),<br>Anti-NeuN antibody, MAB377, Millipore,<br>goat anti-mouse secondary antibody with Alexa Fluor 488, Thermo Fisher, Cat No. A32723<br>rabbit anti-polyGP, AB1358, Millipore,<br>mouse anti-C9orf72 antibody GT779, GeneTex Inc, Cat. No. GTX632041<br>DyLight 594 goat anti-rabbit antibody, Thermo Fisher, Cat. No. 35560
Validation	GT779: Knockdown/Knockout validation was supported by customer review data (supplier website).<br>MAB377: multiple references on supplier website (PMIDs: 24748560, 25330953, 24723034, 24911405, 26450683, 25723967, 25915831, 26140685, 25522418, 25757085).<br>AB1358: references on supplier website, Dürnberger G, Burckstummer T, Huber K, Giambruno R, Doerks T, Karayel E, et al. Experimental characterization of the human non-sequence-specific nucleic acid interactome. Genome Biol. 2013;14:R81;<br>Li Y, Zhou H, Xing E, Dassarath M, Ren J, Dong X, et al. Contribution of decreased expression of Ku70 to enhanced radiosensitivity by sodium butyrate in glioblastoma cell line (U251). J Huazhong Univ Sci Technolog Med Sci. 2011;31:359;<br>Benadiba M, Miyake J, Colquhoun A. Gamma-linolenic acid alters Ku80, E2F1, and bax expression and induces micronucleus formation in C6 glioma cells in vitro. IUBMB Life. 2009;61:244-51.<br>11093274910: multiple references on supplier website, Tara C Carlisle and Angeles B RiberaThe Journal of comparative neurology, 522(4), 861-875 (2013-8-14); Lina K Sciesielski et. al FEBS letters, 584(22), 4665-4671 (2010-10-27); Yoshihiro Komatsu et. al Methods in molecular biology (Clifton, N.J.), 1092, 1-15 (2013-12-10)<br>35560: multiple references on supplier website, Hepatology (Baltimore, Md.) Actin-like 6A predicts poor prognosis of hepatocellular carcinoma and promotes metastasis and epithelial-mesenchymal transition;<br>Scientific reports Down regulated lncRNA MEG3 eliminates mycobacteria in macrophages via autophagy;<br>Cancer medicine Beta emitters rhenium-188 and lutetium-177 are equally effective in radioimmunotherapy of HPV-positive experimental cervical cancer.

Eukaryotic cell lines

Policy information about cell lines

Cell line source(s)	Cos-7: ATCC;<br>iPSCs: patient fibroblasts came from a chromosome 9 open reading frame 72 (C9orf72)-associated female patient with amyotrophic lateral sclerosis (ALS) (64 years old, RUCDR Infinite Biologics);<br>C9-ALS primary fibroblasts: generated from patient fibroblasts;<br>primary cortical neurons: generated from E15.5 C9-BAC transgenic embryos<br>healthy iPCS motor neurons (Lot no. 400238-1M, iXcells Biotechnologies, San Diego, CA),<br>TDP43 M337V iPSC motor neurons ((Lot no. 400311-MN-TDP43HETM337V-CR8-C1, iXcells Biotechnologies, San Diego, CA)
Authentication	ALS-derived iPSCs and primary fibroblasts were validated by Southern blot and transcript profile assessments. Cos-7 cells were received from ATCC and were not authenticated.
Mycoplasma contamination	Cell lines are negative for mycoplasma contamination. iPSC-derived cells were tested by iXcells Biotechnologies using protocol described in Young et al., Detection of Mycoplasma in cell cultures. Nat Protocols 5(5): 929-934 2010.
Commonly misidentified lines<br>(See ICLAC register)	None

Animals and other organisms

Policy information about studies involving animals; ARRIVE guidelines recommended for reporting animal research

Laboratory animals	C9BAC transgenic mice [Tg(C9orf72_3) No. 023099, Jackson Laboratories]; we selected mice expressing ≥500 repeats that were 10-12 weeks old. We utilized both male and female mice.<br>C57BL/6J animals (No. 000664, Jackson Laboratories)
Wild animals	None

Field-collected samples	None
Ethics oversight	All animal experiments were performed at Biomere Biomedical Research Models (Worcester, MA) in compliance with Biomere's Institutional Animal Care and Use Committee guidelines for care and use of animals.

Note that full information on the approval of the study protocol must also be provided in the manuscript.

Human research participants

Policy information about studies involving human research participants

Population characteristics	C9-2. RB19842 were obtained from a 64-year old, Caucasian female; C9-3. RB19895 were obtained from a 59-year old Caucasian male. Both subjects had confirmed C9orf72-repeat expansions and met diagnostic criteria for ALS.
Recruitment	*Describe how participants were recruited. Outline any potential self-selection bias or other biases that may be present and how these are likely to impact results.*
Ethics oversight	UMass Medical School - IRB-approved protocol for skin biopsy and fibroblast culture (IRB No. 13019)

Note that full information on the approval of the study protocol must also be provided in the manuscript.
